# Classification of rice (*oryza sativa *l. japonica nipponbare) immunophilins (fkbps, cyps) and expression patterns under water stress

**DOI:** 10.1186/1471-2229-10-253

**Published:** 2010-11-18

**Authors:** Jun Cheul Ahn, Dae-Won Kim, Young Nim You, Min Sook Seok, Jeong Mee Park, Hyunsik Hwang, Beom-Gi Kim, Sheng Luan, Hong-Seog Park, Hye Sun Cho

**Affiliations:** 1 Plant Systems Engineering Research Center, Korea Research Institute of Bioscience and Biotechnology, Daejeon 305-506, Korea; 2 Genome Research Center, Korea Research Institute of Bioscience and Biotechnology, Daejeon 305-306, Korea; 3 Department of Biological Science, Seonam University, Namwon 590-711, Korea; 4 Bio-crops Development Division, National Academy of Agricultural Science, RDA, Suwon, Korea; 5 Department of Plant and Microbial Biology, University of California, Berkeley, California 94720, USA

## Abstract

**Background:**

FK506 binding proteins (FKBPs) and cyclophilins (CYPs) are abundant and ubiquitous proteins belonging to the peptidyl-prolyl *cis/trans *isomerase (PPIase) superfamily, which regulate much of metabolism through a chaperone or an isomerization of proline residues during protein folding. They are collectively referred to as immunophilin (IMM), being present in almost all cellular organs. In particular, a number of IMMs relate to environmental stresses.

**Results:**

FKBP and CYP proteins in rice (*Oryza sativa *cv. Japonica) were identified and classified, and given the appropriate name for each IMM, considering the ortholog-relation with *Arabidopsis *and *Chlamydomonas *or molecular weight of the proteins. 29 FKBP and 27 CYP genes can putatively be identified in rice; among them, a number of genes can be putatively classified as orthologs of *Arabidopsis *IMMs. However, some genes were novel, did not match with those of *Arabidopsis *and *Chlamydomonas*, and several genes were paralogs by genetic duplication. Among 56 IMMs in rice, a significant number are regulated by salt and/or desiccation stress. In addition, their expression levels responding to the water-stress have been analyzed in different tissues, and some subcellular IMMs located by means of tagging with GFP protein.

**Conclusion:**

Like other green photosynthetic organisms such as *Arabidopsis *(23 FKBPs and 29 CYPs) and *Chlamydomonas *(23 FKBs and 26 CYNs), rice has the highest number of IMM genes among organisms reported so far, suggesting that the numbers relate closely to photosynthesis. Classification of the putative FKBPs and CYPs in rice provides the information about their evolutional/functional significance when comparisons are drawn with the relatively well studied genera, *Arabidopsis *and *Chlamydomonas*. In addition, many of the genes upregulated by water stress offer the possibility of manipulating the stress responses in rice.

## Background

Proteins that bind to immunosuppressive drugs, such as FK506, rapamycin and cyclophilin A (CsA), have been called FKBPs (FK506/rapamycin-binding proteins) and CYPs or cyclophilins (cyclosporin A-binding proteins), respectively, being collectively referred to immunophilins [1]. Despite their lack of structural similarity, these two families share a common peptidyl-prolyl isomerase (PPIase), catalyzing the *cis/trans *isomerization of proline imidic peptide bonds [2]. The *Cis/trans *isomerization of the Xaa-Pro bond results in slow phases in protein folding, which is an important step for folding and a critical determinant of structure [3].

Biochemical analysis and sequence analysis following genome sequencing projects have identified a large number of IMMs and, in particular, putative IMMs in many organisms [4-6]. As a result, IMMs are highly conserved ubiquitous proteins found in most organisms and in all major subcellular compartments. However, the number of IMMs in different organisms differs greatly. For example, some prokaryotes do not contain any FKBPs or CYPs, and some prokaryotes encode only one family of ribosome-associated PPIase known as trigger factors (TIFs), which aid in the folding of nascent polypeptide chains on ribosomes [7]. *Escherichia coli *contains 6 IMMs, the yeast genome contains 12 (4 genes for FKBPs and 8 genes for CYPs), *Drosophila melanogaster *contains 21 ones (7 FKBPs and 14 CYPs), and *Caenorhabditis elegans *contains 25 (8 FKBPs and 17 CYPs). The human genome contains 42 (18 FKBPs and 24 CYPs). Notably, photosynthetic organisms harbor a remarkably large number of IMMs, with 52 genes (23 FKBPs and 29 CYPs) in *Arabidopsis*, 49 (23 FKBPs and 26 CYPs) in *Chlamydomonas*, and 29 FKBPs in rice [4,6,8,9]. The greater numbers of IMMs in green photosynthetic organisms was predicted to be due to the largest IMM family targeted to the photosynthetic apparatus [4,10]. For example, 11 FKBPs and 5 CYPs localize to the chloroplast thylakoid lumen in *Arabidopsis*, creating the largest IMM family in any cellular organism.

Despite the high level of conservation of the PPIases throughout eukaryotes and prokaryotes, they do not have a function within many cells under normal growth conditions, but may become essential in the absence of other cellular factors [5]. Noteworthy are the differences in the phenotypical consequences for microorganisms and higher organisms in response to the deletion of individual PPIase genes. Most strikingly, of all genes encoding FKBPs and CYPs, a total of 12 can be deleted without seriously affecting the viability of *Saccharomyces cerevisiae *[11]. However, higher organisms are seriously affected even in their response to a single gene deletion. For example, *FKBP12-/- *mice suffer early embryonic death due to cardiac defects, and the disruption of genes that encode for the nuclear-localized FKBP42 and FKBP70 leads to multiple morphogenetic defects in *Arabidopsis thaliana *[12,13]. Although dramatic changes in the phenotypes of PPIase-deficient higher organisms are very limited, they suggest that IMMs have been evolving toward highly specialized functions from microorganisms to higher organisms.

The expression of archetypical IMM genes has some typical characteristics of housekeeping genes that encode proteins required for general functioning [14]. On the other hand, the expressions of certain IMM genes are upregulated under stress conditions. Expression of some IMMs has been induced by both biotic and abiotic stresses, including HgCl_2_, viral infection, salicylic acid, salt stress, heat and cold shock, light and drought [15-21]. Furthermore, the large number of IMMs located in the thylakoid lumen in *Arabidopsis *is upregulated only by light conditions; analysis of the microarray data showed that the expression of several IMM genes was regulated by certain environmental conditions [4].

Rice has been cultivated as a major crop for > 7000 years, and currently sustains more than half of the world's population [22]. Because rice is a semi-aquatic plant commonly grown under flooded conditions, production in Asia has been dramatically increased as a result of the "Green Revolution" following the high-input of irrigation systems since 1961 [23]. However, about half the rice-growing areas in the world do not have sufficient water to maintain flooded conditions, and yield is also reduced to some extent by various abiotic stresses, including drought and salinity. Plant breeders in the global research group have repeatedly developed more productive drought-tolerant and salt-resistant rice. As a result, even where the response to stress in rice is better than that of other crops, many rice-growing environments demand still greater tolerance than can be found in most improved germplasm. In addition, rice has the smallest genome size among cultivated cereals, and it conserves much of the gene content, and to some extent the gene order, present in other species. The full rice genome has now been sequenced, allowing the identification and localization of genes related to stress tolerance [24].

We have identified 2 rice IMM families, FK506 binding proteins and cyclophilins, using the rice genome sequence database. We have given appropriate names to individual rice IMM (Os IMM) based on its ortholog relation with *Arabidopsis *(At) and *Chlamydomonas *(Cr) IMMs and the molecular weight of the proteins. In addition, a comprehensive analysis of rice IMM FKBPs and CYPs, including putative domain architecture, amino acid alignment, the conservation ratio of key amino residues for binding of immunosuppressive drugs, and phylogenetic relationships between family members, was also conducted. Furthermore, to select water stress-related IMMs from among the putatively classified rice IMM genes, expression patterns of all IMMs under salt and desiccation stress conditions were analyzed. IMMs responding to water stress were investigated in different tissues, and the subcellular localization some IMMs were investigated by tagging with GFP protein.

Detailed information about the 2 major IMM family FKBPs and CYPs, as well as information about several IMM genes probably related to water stress in the case of rice, can provide new possibilities for stress research related to crop plants, given that rice and many other crop plants are monocotyledons that are distinct from the dicotyledonous model plant, *Arabidopsis*, in many aspects of their development.

## Results and Discussion

### Identification and Nomenclature of Immunophilin Genes in the *O. sativa *Genome

In an attempt to detect IMM genes in the rice genome, we searched the GRAMINE database. As a result, 56 putative IMM genes, consisting of 29 putative FKBPs and 27 putative CYPs, were identified. Additional file 1 shows the 56 genes with their names, accession numbers, putative subcellular localization, isoform numbers, amino acid numbers, number of ESTs, molecular weight, pI, expression level, and similarities with orthologous genes in *Arabidopsis thaliana *and *Chlamydomonas reinhardtii *IMMs. Like other photosynthetic organisms encoding the putative 52 genes of *Arabidopsis*, 49 genes of *Chlamydomonas*, putative 27 genes for FKBPs and 36 genes for CYPs of *Sorghum bicolor*, and putative 26 genes for FKBPs and 25 genes for CYPs of *Vitis vinifera *(our unpublished data), rice also has one of the largest IMM families in organisms whose genomes have been completely sequenced [4,6]. In addition, considering that alternative splicing occurs as a normal phenomenon in eukaryotes, the diversity of IMM proteins encoded by a genome may also increase [25]. In rice, 12 genes of OsFKBPs and 14 genes of OsCYPs may encode alternatively spliced isoforms ranging from 2 to 4 as (Additional file 1), but it is not to purpose of this paper to provide the complete number of IMM proteins functioning in rice. More to the point, *Escherichia coli *possesses 6 IMMs, including 3 FKBPs and 2 cyclophilins. The yeast *Saccharomyces cerevisiae *contains 4 FKBPs and 8 CYPs. The soil nematode *Caenorhabditis elegans *genome contains 8 genes for FKBPs and 24 genes for CYPs. The human genome contains 18 genes for FKBPs and 24 genes for CYPs. A large number of IMMs in eukaryotes and, in particular, in plants remains evolutionarily and functionally obscure. However, many IMMs in plants may serve a more diverse array of functions, especially in relation to photosynthesis, but may also involve a significant amount of redundancy [4].

For the nomenclature of rice IMMs, we followed the previously reported rule for *Arabidopsis*: the proteins were named FKBP for the FKBP506-binding protein and CYP for CsA, with prefix letters to indicate the species of origin (e.g., Os for *Oryza sativa*) and a suffix number to indicate *M*_r_. For genes where > 2 protein isoforms can arise from the same gene by alternative splicing, only the longest form was used in this nomenclature [4]. Where possible in considering characteristics such as > 50% identity in homology, the same subcellular location and the same domain architecture as with *Arabidopsis *and *Chlamydomonas *IMM proteins, we used the same suffix numbers for genes whose orthologs could be deduced. These include OsFKBP12, -13, -15-1, -15-2, -16-1, -16-2, -16-3, -16-4, -17-1, -17-2, -18, -19, -20-2, -53, and -72 for FKBPs, and OsCYP18-1, -18-2, -18-4, -19-2, -19-3, -19-4, -20-1, -20-2, -20-3, -21-1, -21-4, -22, -23, -26-2, -28, -37, -38, -57, -63, -65, -71, and -95 for CYPs. For some genes where > 2 genes show an orthologous sequence with the same protein as *Arabidopsis and Chlamydomonas*, lowercase extension letters were added to the name according to the order of similarity. These were OsFKBP20-1a, -20-1b, -42a, -42b, -62a, -62b, and -62c for FKBPs and OsCYP40a, -40b, -59a, and -59b for CYPs. For other genes whose orthologs in *Arabidopsis *or *Chlamydomonas *were not obvious, these were named after the calculated *M*_r _of mature proteins predicted from cDNA-deduced sequences. Here, OsFKBP44, -46, -57, -58, -59, and -73 for FKBPs, and OsCYP17 for CYPs were included. One gene encoding trigger factor-like protein, distantly related to the FKBP family, was found in the rice genome, as in *Arabidopsis*, and was named OsTIG. As for the nomenclature of the rice FKBP family, there has been a preceding report in which almost all the nomenclature agreed with our nomenclature; however, we substituted OsFKBP53b and -53a by Os FKBP53 and -58, respectively, and also OsFKBP64, -65 and -75 by OsFKBP62a, -62b and -62c, re-spectively. The reason for this is discussed below [4,8,26].

### Rice FKBP-506 Binding Proteins (OsFKBPs)

**Figure 1:**
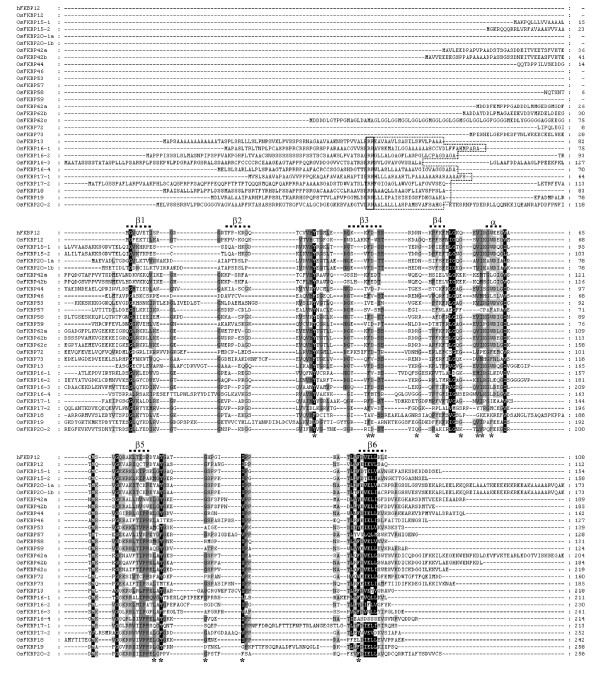
**Multiple sequence alignment of *O. sativa *FKBPs**. Human FKBP12 (hFKBP12, GeneBank accession no. A35780) was used for determined for hFKBP12 were marked by asterisks (*). Secondary structure features (a-helix and b-sheets) derived from the hFKBP12 were displayed. The targeting signal of putative chloroplast (thylakoid lumen) localized OsFKBPs was manually adjusted (boxed): double Arg residues (--); the hydrophobic stretches following the double Args (---). Amino acids of full length protein for single FKBP domain OsFKBPs, and only the most conserved amino acids area for multiple FKBP domain OsFKBPs, were aligned. Backgrounds indicate percentage of amino acid similarity: black, 95%; dark grey, 70%; light grey, 40%.

**Figure 2:**
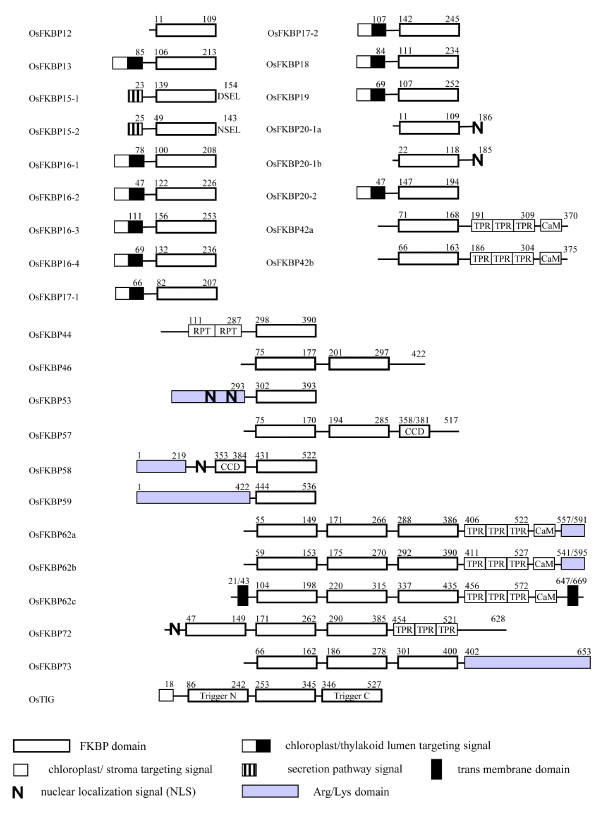
**Domain architecture of the *O. sativa *FKBP immunophilins**. The beginning and ending amino acids numbers of each protein are shown at each end of the diagram. FKBP domains are represented by white box. The other functional domains such as tetrapeptide repeats (TPR), Arg/Lys amino acids rich domain (gray background box), coiled coil domain (CCD), internal repeats (RPT) and Calmodulin-binding motif (CaM) are indicated separately. The transmembrane domains (TM) that are not part of the signal peptide are labeled. The bipartite chloroplast/thylakoid targeting signal, ER Targeting signal, and the NLS are represented. The numbers above the boxes denote the amino acid positions of the function domains.

**Figure 3:**
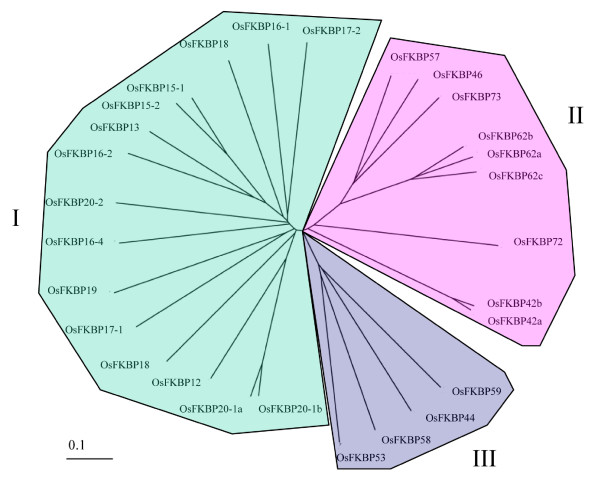
**Unrooted phylogenetic trees of *O. sativa *FKBP proteins**. The sequence alignment of Figure 1 (OsFKBPs) was used and the phylogenetic analysis was based on the sequence alignments by ClustalX http://align.genome.jp.

To compare the conservation patterns of the amino acid residues for the binding of immunosuppressive drug (ISD) FK506/PPIase activity and secondary structural details, the amino acids of 29 OsFKBPs were co-aligned with human FKBP12 (hFKBP12) as an external reference. hFKBP12 identifies the 14 amino acid residues for the FK506 binding and PPIase activity [27-29]. For FKBPs with a multiple (two or more) FKBP domain, the most conserved domain obtained from the analysis using the SMART program was used (Figure 1). In addition, to analyze conservation in amino acids for FKBP domain and their orthologous relationship, the conservation patterns and ratio of the amino acid residues for FKBP domain between each ortholog of rice and *Arabidopsis *FKBPs was compared (Table 1). The 29 OsFKBPs identified could be classified into single-domain (SD) members with a FKBP catalytic domain, and multiple-domain (MD) members with other functional domains in addition to a single or multiple FKBP domains (Figure 2). The functional domains include tetra-peptide repeats (TPR), the coiled-coil domain (CCD), and the internal repeats domain (RPT), each of which is involved in protein-protein interactions or recognitions, or in the assembly of multi-protein complexes [30,31]. The Arg/Lys amino acid-rich domain may function as a motif for non-specific RNA binding or mediate protein-protein interactions, and is also a frequent target for molecular interactions [32-34]. The calmodulin-binding motif (CaM) has been recognized as a major Ca^2+ ^sensor and as a modulator of regulatory events through its interaction with a protein [35]. Finally, the full length of the amino acid sequences of all FKBPs was used to generate a phylogenetic tree in order to assay the evolutionary relationships among the OsFKBPs (Figure 3).

**Table 1:** Conservation of key residues for FK506 binding/PPIase activity in *O. sativa *FKBPs (OsFKBPs) and comparison with those of *A. thaliana *FKBPs.

OsFKBPs/AtFKBPs		Residues for FK506 binding/PPIase activity in human FKBP12 (aa position)														Conserved^a ^(%)	Similarity^b^ (%)
				
		27	37	38	43	47	55	56	57	60	82	83	88	92	100		
hFKBP12		Y	F	D	R	F	E	V	I	W	A	Y	H	I	F		
OsFKBP12	: AtFKBP12	C	"	W	A/E	"	S/A	"	"	"	"	"	F	"	"	64/64	86
OsFKBP13	: AtFKBP13	"	"	"	"	L	"	"	"	"	"	"	A	"	"	86/86	100
OsFKBP15-1	: AtFKBP15-1	"	"	"	"	I	Q	"	"	"	G	"	S	"	"	71/71	100
OsFKBP15-2	: AtFKBP15-2	"	"	"	"	F	Q	"	"	"	G	"	S	"	"	71/71	100
	: AtFKBP15-3	"	"	"	K	Y	K	"	"	L	G	"	-	"	"	-/57	
OsFKBP16-1	: AtFKBP16-1	"	V	H	Q	V	"/D	M/V	"	L	G	"	P	L	"	36/29	86
OsFKBP16-2	: AtFKBP16-2	"	"	"	"	L	K	I/"	L/"	L	"	"	G	"	Y	50/64	86
OsFKBP16-3	: AtFKBP16-3	"	"	"	K	Y	Q	"	"	L	"	F	A	V	"	50/50	100
OsFKBP16-4	: AtFKBP16-4	"	"	M	G	Y	"	"	L	L	"	"	V	A/L	"	50/50	93
OsFKBP17-1	: AtFKBP17-1	"	"	"	H	"	N/K	"	"	I	G	"	Q	P	"	57/57	93
OsFKBP17-2	: AtFKBP17-2	L	V	V	G/K	L	P	Y	T/S	M/L	G	F	A	V/"	Y	0/7	71
	: AtFKBP17-3	V	V	"	K	L	P	Y	S	L	G	F	Q	"	Y	-/14	
OsFKBP18	: AtFKBP18	F	L	A	I	Y	P	G	K	K	G	"	M	"	L	14/14	100
OsFKBP19	: AtFKBP19	W	"	E	D	"	Q/"	"	"	F	G	"	Y	G	"	43/50	93
OsFKBP20-1a	: AtFKBP20-1	"	"	"	D	"	A/S	"	"	"	"	"	S	"	"	79/79	93
OsFKBP20-1b		"	"	"	D	"	T	"	"	"	"	"	S	"	"	7/-	
OsFKBP20-2	: AtFKBP20-2	"	I	"	Q	I	T/A	L	V	F	G	P	P	F	"	21/21	93
OsFKBP42a	: AtFKBP42	"	"	E	E	I	K	E	M/L	L	G/"	"	S	V	Y	21/29	86
OsFKBP42b		"	"	E	E	I	K	Q	M	L	G	"	S	V	Y	21/29	
	: AtFKBP43	"	"	"	E	L	N	"	"	L	G	"	G	K	Y	-/43	
OsFKBP44		"	V	"	S	C	"	"	"	"	G	"	R	"	"	64/-	
OsFKBP46		F	"	V	N	"	N	"	M	L	"	I	S	"	"	43/-	
OsFKBP53	: AtFKBP53	"	V/"	E/"	E/K	"	K/S	"	"	"	C/G	"	I/A	V/"	Y/"	43/64	44
OsFKBP57		"	V	S	-	I	F	F	C	F	G	F	R	V	V	7/-	
OsFKBP58		"	"	"	"	"	"	"	"	"	G	"	R	"	"	79/-	
OsFKBP59		"	I	"	N	H	"	"	"	"	G	"	T	"	Y	57/-	
OsFKBP62a	: AtFKBP62	"	"	"	"	"	Q	"	"	"	"	"	S	"/-	"	86/79	93
OsFKBP62b	: AtFKBP62	"	"	"	"	"	"/Q	"	"	"	"	"	S	"/-	"	93/79	86
OsFKBP62c	: AtFKBP62	"	"	"	"	"	Q	"	"	"	"	"	S	"/-	"	86/79	93
	: AtFKBP65	"	"	"	"	"	H	"	"	"	"	"	S	-	"	-/79	
OsFKBP72	: AtFKBP72	"	Y	"	N	"	L	"	P	F	"	"	R	V	W	43/43	100
OsFKBP73		F	"	V	N	E	D	"	M	F	T	M	S	"	"	36/-	
Conserved^c ^(%)		79/77	68/73	57/64	32/27	43/36	25/14	75/73	68/68	46/36	46/46	82/82	0/0	46/50	64/64		

^a^% identity of rice/*Arabidopsis *FKBP compared to hFKBP12. ^b^% similarity between rice and *Arabidopsis *FKBP. ^C^% conservation of amino acid in rice/*Arabidopsis *FKBP

The conservation patterns of the amino acid residues for the binding of ISD showed great differences between OsFKBPs (Figure 1, Table 1). For example, some OsFKBPs (OsFKBP13, -15-1, -15-2, -20-1a, -20-1b, -58, -62a, -62b, and -62c) share identities of > 70%, with 14 key residues of hFKBP, whereas other OsFKBPs (OsFKBP16-1, -17-2, -18, -20-2, -42a, -42b, and -57) share identities to < 30%. In the case of OsFKBP17-2, all key residues are substituted by other residues. The conservation patterns of OsFKBPs with the 14 amino acid residues of hFKBP12 were very similar to those of the *Arabidopsis *(Table 1) and *Chlamydomonas *orthologs [6]. However, the repertoire and amino acid sequence of rice FKBPs are more similar to those of *Arabidopsis *than *Chlamydomonas*, from which they have a distant genetic distance. Moreover, the diversity of IMM proteins was somewhat different for the monocot rice than for the dicot *Arabidopsis*. This information added broader evidence in favor of the theory that the diversity of IMMs was already established in their last common ancestor, probably close to the root of the "green" lineage of plants, but they may have continued to change and become more diverse since the completion of the "green" lineage. In particular, the conservation between FKBPs, as in the FKBP repertoire, among *O. sativa*, *Arabidopsis *and *Chlamydomonas *was lower than in cyclophilins (see below), as reported in a comparative analysis of various organisms containing *H. sapiens, D. melaogaster, C. elegans, S. cereviseiae and Sz. Pombe *[5,6].

Of the 29 FKBPs containing OsTIG, 15 have been characterized as SD members. All these proteins contain a single FKBP domain, and some harbor a targeting sequence that determines the subcellular localization of the mature protein (Figure 2). OsFKBP12 is the only cytosolic FKBP having SD members, as in *Arabidopsis*. It features a possible role for the regulation of the cell cycle through the interaction with AtFIP, a phosphatidyl-inositol kinase [36]. Moreover, it was also expressed strongly (Additional file 1) as in *Arabdopsis *AtFKBP12 and *Chlamydomonas *FKB12, with the largest number of ESTs [4,6].

Ten OsFKBPs (OsFKBP13, -16-1, -16-2, -16-3, -16-4, -17-1, -17-2, -18, -19, and -20-2) contain putative chloroplast-targeting sequences in the N-terminal region. The signal sequences of all chloroplast FKBPs have double arginine residues and follow the hydrophobic region needed for chloroplast lumen translocation via the Tat (twin arginine translocase) pathway [37]. The majority of chloroplast FKBPs have a peptidase cleavage site, Ala-Xaa-Ala, at the terminus of the hydrophobic region (Figure 1), suggesting that all the chloroplast FKBPs target the thylakoid lumen like the chloroplast FKBPs of *Arabidopsis and Chlamydomonas*. However, like orthologs of *Arabidopsis*, AtFKBP17-2, -18, and -20-2 also lack a cleavage site at the terminus of the hydrophobic extension region, suggesting that they act as insoluble proteins within the thylakoid lumen, but proteins anchored in the thylakoid membrane [4]. Compared with *Arabidopsis*, in which the thylakoid lumen FKBPs have two FKBPs (AtFKBP17-2 and 17-3) with high sequence similarity, all thylakoid lumen FKBPs are well conserved in both rice and *Arabidopsis*, except in the absence of OsFKBP17-3 in rice, considering the domain architecture (Figure 2), the alignment of each amino acid sequence between those with two (data not shown), and the high similarity of conserved key amino acid residues for ISD binding (Table 1). Thus, AtFKBP17-2 and 17-3, seen as gene duplicates based on their high sequence similarity, have been duplicate since the divergence of the evolutionary lineage between rice and *Arabidopsis*. The common ancestor of the eukaryotic green lineage, *Chlamydomonas*, also has 11 FKBPs (FKB), but orthology among them can only be claimed between FKB16-3, -16-4, -18, -19, and 20-2 and the *Arabidopsis *genes with the same numbering. For the others, it has been difficult to match the ortholog relations [6]. All in all, the diversity of chloroplast lumen FKBPs has been established in *Chlamydomonas *based on the number of IMMs.

Two OsFKBPs (OsFKBP15-1 and OsFKBP15-2) contain putative ER targeting sequences at the N-terminus and have possible C-terminal endoplasmic reticulum (ER) retention signals (DSEL and NSEL for OsFKBP15-1 and OsFKBP15-2, respectively) (Figure 1 and 2), although it will need to be confirmed whether they function as real anchors in the ER membrane. They are closely related in sequence, and they are 81% similar to AtFKBP15-1 and 76% similar to AtFKBP15-2 in their amino acid sequences (Additional file 1).

Two OsFKBPs (20-1a and 20-1b) are characterized as putative nuclear targeting paralogous proteins by the presence of nuclear localization signals (NLS) in the C-terminal region (Figure 2). They are also closely related in their amino acid sequence (Figure 1), and are orthologous to AtFKBP20-1, with 77 and 76% similarity in their amino acid sequences, respectively (Additional file 1). Interestingly, *Arabidopsis *has only single AtFKBP20-1 and AtFKBP15-3, and another nuclear targeting SD FKBP has its signal at the N-terminal region, whereas rice lacks an ortholog for AtFKBP15-3 [4].

14 OsFKBPs containing OsTIG belong to MD members. Among them, two isoforms (a/b) of OsFKBP42 are characterized by a single FKBP domain, TPR domain, and CaM domain (Figure 2). However, unlike with orthologs *Arabidopsis *AtFKBP42 and *Chlamydomonas *FKBP42 that have a membrane-anchoring domain at the C-terminus, and also contrary to a previous report on OsFKBP42a/b, we could not find a C-terminal membrane-anchoring domain at either OsFKBP42a or -42b using various prediction programs found on the ExPASY web site http://www.expasy.ch/tools[4,6,8]. In reality, the amino acids at the C-terminus in which the membrane-anchoring domain is positioned showed significant changes between AtFKBP42 and OsFKBP42a/b (data not shown). It is therefore supposed that the anchoring of this protein to the tonoplast or plasma membrane may be not essential for the functioning of the protein, unlike with AtFKBP42 that was predicted to be membrane-localized [38]. OsFKBP42a (LOC_Os12g05090) and OsFKBP42b (LOC_Os11g05090) are positioned in terminal regions of chromosome 11 and 12 in rice that are known to result from a duplication 7.7 million years ago, the most recent large-scale duplication in the rice genome [39].

Other MD FKBPs with a TPR domain are OsFKBP62a, -62b, and -62c. These three genes were previously named *OsFKBP64, -65*, and *-75 *and *rFKBP64, -65*, and *-75*, respectively, based on their molecular weight and similarity to two MD AtFKBP62 and -65 with triple FKBP domains, a TPR domain, and a CaM domain in common [4,8,40]. However, OsFKBP62a, -62b, and -62c retain 77, 75 and 73% similarity with AtFKBP62 in amino acid sequence, respectively, and 41, 41 and 42% similarity with FKB62, respectively (Figure 2, Additional file 1), and all belong to one stem branch of clade II in the phylogenetic tree (Figure 3). In addition, these three proteins have almost the same 14 key residues for PPIase activity and are conserved in 12 out of 14 key residues of hFKBP12 for PPIase activity, suggesting that they are catalytically active isoforms (Table 1). AtFKBP62 and -65 were thought to have originated from regional duplicates between chromosomes I and V [4]. Taken together, all this suggests that rice MD OsFKBP62a, -62b and -62C are paralogs duplicated from an ancestral gene, probably *Chlamydomonas *FKB62, whereas *Arabidopsis *has only two duplicated paralogs, AtFKBP62 and -65. In the meantime, unlike OsFKBP62a and -62b, OsFKBP62c (OsFKBP75, rFKBP75) contains ~50 residues of a glycine repeat domain in the N-terminal area and an extension of ~40 residues at the C-terminus, identified to the putative transmembrane domain (Figure 2). The glycine repeat domain at N-terminus is associated with consensus targeting for the endoplasmic reticulum, and rFKBP75 was predicted to be an ER membrane protein [40]. In *Arabidopsis*, AtFKBP62 (ROF1) and AtFKBP65 (ROF2) are regulated by age and biotic stresses [19,41,42]. Recently, ROF1 was thought to have played a role in the prolongation of thermotolerance by sustaining the levels of small HSPs essential for survival at high temperatures. Additionally, the ROF1-HSP90.1 complex was localized in the nucleus during exposure to heat stress, whereas it was localized in the cytoplasm under normal conditions [19].

OsFKBP72 - with a triple FKBP domain, TPR domain, and NLS at the N-terminal area - shows 67% similarity and absolute similarity in terms of amino acid residues for PPIase with AtFKBP72 (Figure 2, Table 1). Unlike other MD FKBPs, which are composed of triple FKBP domains, the second FKBP domain of OsFKBP72 is the most highly conserved domain and shows remarkable differences in terms of key residues of hFKBP12 for ISD binding/PPIase activity. It also forms a separate branch within clade II in the phylogenic tree (Figure 3), suggesting that it originated from another ancestral gene and is likely to lose its function as PPIase. AtFKBP72 (PAS1) is involved in the control of cell proliferation and differentiation during plant development [43,44]. The C-terminus of PAS1 is required for binding with the NAC-like transcription factor and nuclear translocation [44]. OsFKBP72 also shows considerable similarity with AtFKBP72 in the C-terminal area. However, we could not find the CaM domain C-terminal area of OsFKBP72 using a CaM prediction program, unlike with AtFKBP72 (PAS1), a putative CaM domain in the C-terminal area due to the binding of calmodulin *in vitro *[43].

Among the MD FKBPs belonging to clade II, OsFKBP73 with its triple FKBP domain and OsFKBP46 and OsFKBP57 with their double FKBP domains diverge within the same branch node and show partial similarities in terms of key residues for ISD binding/PPIase (Figure 3, Table 1). OsFKBP73 has a long Arg/Lys amino acid-rich domain (from aa 402 to 653) at the C-terminus, and OsFKBP57 retains a coiled coil domain in the C-terminal area (Figure 2). OsFKBP46 and OsFKBP57 that have no signal motifs were predicted to be cytosolic, whereas OsFKBP73, with its long Arg/Lys amino-acid-rich domain, was predicted to be nuclear by several prediction programs. Otherwise, as in the case of PAS1, we infer that it is a cytosol/nucleus shuttle protein. Orthologs of these three FKBPs are unlikely to be in *Arabidopsis*, although they have weak identity with AtFKBP65 in their amino acid sequences (Additional file 1). Also, the putative orthologs of these genes that are highly conserved in amino acid sequence existed in other plants containing *Poplus *and *Vitis *(data not shown). The novelty of these genes and the suggestion that these three genes originated from the regional duplications of rice chromosome 1 has recently been reported [8].

In clade III, four MD FKBPs (OsFKBP44, -53, -58, and -59) were grouped as 4 branches of the same node (Figure 3) and were analyzed to the highest identity with AtFKBP53 and FKB53 (Additional file 1) via blastP analysis. Among them, OsFKBP53 and -58 contain highly charged domains, putative nuclear targeting signals in the N-terminal regions, and a single FKBP domain (Figure 2). OsFKBP58 and -53 were previously named OsFKBP53a and -53b, respectively [8]. However, as stated above, all four OsFKBPs of clade III have the closest similarity to AtFKBP53 and FKB53 as a probable ancestral protein. In addition, unlike OsFKBP53a that displays 53% similarity to AtFKBP53, the other three FKBPs display < 50% similarity to AtFKBP53 (Additional file 1). Thus, we named only OsFKBP53 as an ortholog of AtFKBP53, and the others were referred to by molecular weight because there were no adequate orthologs in *Arabidopsis*. OsFKBP58 has 36% similarity to AtFKBP53 (Additional file 1) and retains 8 - 20 mer of amino acid insertion in 3 areas besides the FKBP domain, and thus the CCD domain was predicted to be between amino acids 353 and 381 (Figure 2). OsFKBP44 has a long N-terminal area containing two internal repeat domains and a FKBP domain, showing some degree of similarity to AtFKBP15-3 (data not shown). MD AtFKBP53 and a SD AtFKBP15-3 showed high sequence similarity, but also that rice lacks an ortholog of AtFKBP15-3, suggesting that these proteins (OsFKBP44 and AtFKBP15-3) also originated from *Chlamydomonas *FKB53, but that duplication of two proteins occurred after the lineage divergence of the two plants from a common ancestor.

Finally, the distantly related member of the FKBPs is the trigger factor OsTIG, which also contains an FKBP domain, an N-terminal and C-terminal domains, both of which help to bind ribosomes, probably being located in the chloroplast stroma. The precise function of the trigger factor in the chloroplast remains unknown, but it is a PPIase and chaperone associated with the ribosome that is involved in the early steps of protein folding [45].

### Rice Cyclosporin-A Binding Protein (OsCYPs)

**Figure 4:**
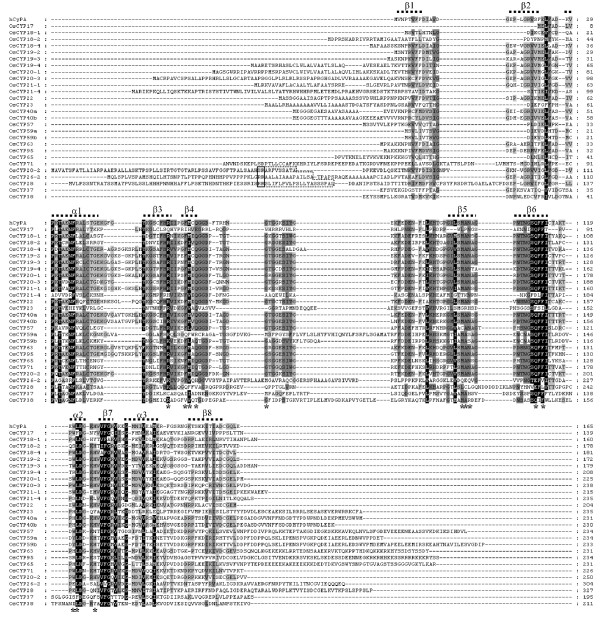
**Multiple sequence alignment of *O. sativa *CYPs**. Human CyPA (hCyPA, GeneBank accession no. P62937) was used for comparison. The amino acids necessary for CsA binding as determined for hCYPA were marked by asterisks (*). Secondary structure features (a-helix and b-sheets) derived from the hCYPA were displayed. The targeting signal of putative chloroplast (thylakoid lumen) localized OsCYPs was manually adjusted (boxed): double Arg residues (--); the hydrophobic stretches following the double Args (---). Amino acids of full length protein for single CYP domain OsCYPs, and only the most conserved amino acids area for multiple CYP domain OsCYPs, were aligned. Backgrounds indicate percentage of amino acid similarity: black, 95%; dark grey, 70%; light grey, 40%.

The 27 identified OsCYPs can be classified as either SD members with a CYP catalytic domain or MD members with other functional domains in addition to a single CYP domain. This number compares well with the 29 AtCYPs for *Arabidopsis *and the 26 genes for *Chlamydomonas*, indicating that the diversity of CYPs has been maintained or even slightly increased since they were established in the photosynthetic green ancestor. To compare the conservation patterns of the amino acid residues for CsA binding/PPIase activity and secondary structural details, the full length of the single domain of CYPs and a truncated CYP domain for the MD CYPs were used for the amino acid alignment. Human cyclophilin A was co-aligned as an external reference (Figure 4), and the full-length of the amino acid sequences of all CYPs was used to generate a phylogenetic tree.

**Figure 5:**
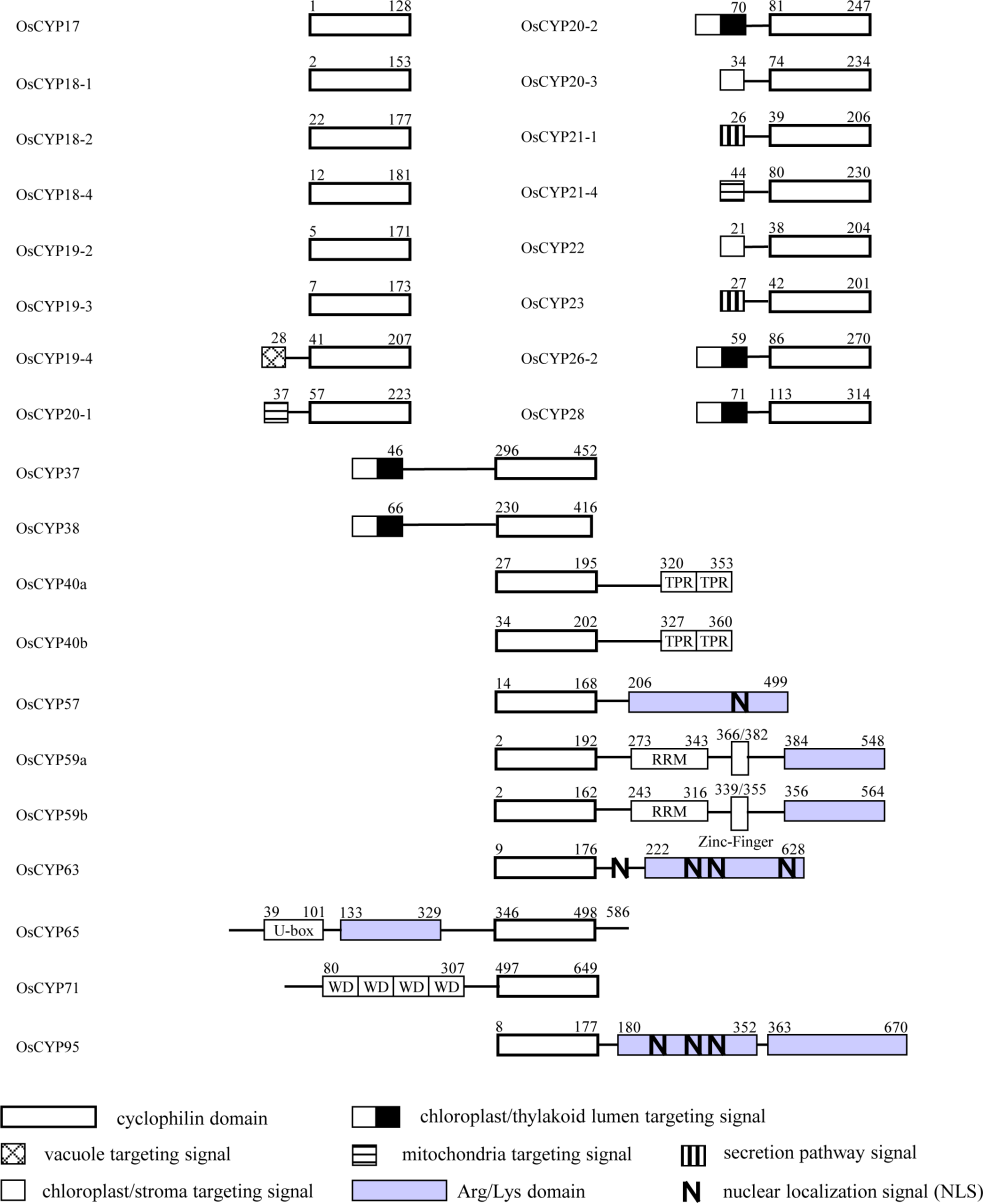
**Domain architecture of the *O. sativa *CYP immunophilins**. The beginning and ending amino acids numbers of each protein are shown at each end of the diagram. CYP domains are represented by white box. The other functional domains such as tetrapeptide repeats (TPR), Arg/Lys amino acids rich domain (gray background box), U-box, WD repeats (WD), RRM, Leu-zipper, and zinc-finger are indicated separately. The bipartite chloroplast/thylakoid targeting signal, ER Targeting signal, and the NLS are represented. The numbers above the boxes denote the amino acid positions of the function domains.

Among the 27 CYPs, 18 are SD members and 9 have been characterized as MD proteins. As with *Arabidopsis*, none of the cyclophilins contains multiple catalytic domains. They also contain more divergent functional domains, such as the TPR domain, the WD-40 repeat, the U-box domain and the Zinc finger, each of which is involved in protein-protein or protein-DNA interactions. An RNA recognition motif (RRM) that may interact with RNA was also found. 6 of the 9 MD CYPs have Arg/Lys amino acid-rich domains that may function as a motif for non-specific RNA-binding or mediate protein-protein interactions, and are also frequent targets of molecular interactions (Figure 5) [32-34].

Based on these data, we assigned the putative one-to-one orthology between *O. sativa *and *Arabidopsis *CYPs. Among 27 OsCYPs, 26 members could have matching orthology with a protein among *Arabidopsis *CYPs, whereas for only OsCYP17, we could find no obvious orthology with *Arabidopsis *CYPs.

**Figure 6:**
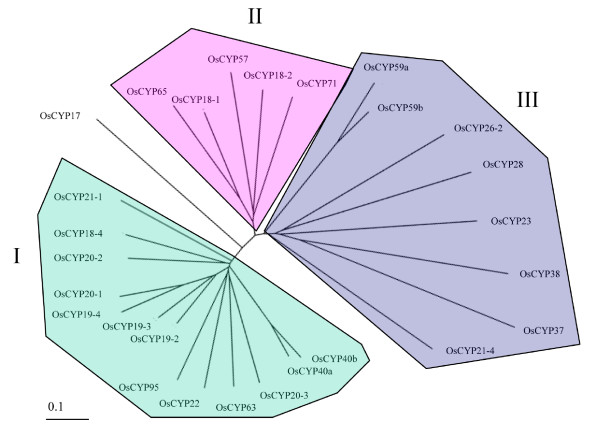
**Unrooted phylogenetic trees of *O. sativa *CYP proteins**. The sequence alignment of Figure 4 (OsCYPs) was used and the phylogenetic analysis was based on the sequence alignments by ClustalX http://align.genome.jp.

6 SD CYPs (OsCYP17, -18-1, -18-2, -18-4, -19-2, -19-3) lack an N-terminal signal peptide and are probably cytosolic. Among the OsCYPs, OsCYP17 is the smallest cyclophilin due to the deletion of β1 and β2 sheets (Figure 4); it retains 37% identity with AtCYP19-1 in amino acid sequences checked with BlastP (Additional file 1). However, OsCYP17 and AtCYP19-1 are significantly different in the conservation ratio of key residues for CsA binding, each of which showed 43 and 100% conservation, respectively (Table 2). In addition, OsCYP17 forms a separate branch within clade I of the phylogenic tree (Figure 6). It is noteworthy that except for *O. sativa *ssp. Japonica and ssp. Indica, no proteins with orthology to OsCYP17 were found in any other plants using BlastP analysis (data not shown). These results suggest that OsCYP17 occurred most recently in the evolution of *O. sativa*. OsCYP18-1 and -18-2 retain 89 and 82% identity with AtCYP18-1 and -18-2 in amino acid sequence, and 70 and 75% with CYN18-1 and -18-2, respectively (Additional file 1). Also, the two proteins exhibit phylogenetic divergence due to a tetra- (18-2) or penta- (18-1) peptide deletion in the β2 domain (Figure 4 and Figure 6). It is suggested that these two proteins may have a distinct and unchanged function for a long period of time, though with a low level of expression (Additional file 1). In addition, these two proteins belong to clade II, which is comprised of the two SD CYPs (OsCYP18-1 and -18-2), as well as 3 MD CYPs (OsCYP57, -65 and -71), and OsCYP18-1 and OsCYP65 share the same branch (Figure 6). These patterns are almost the same in rice, *Arabidopsis*, and *Chlamydomonas*, suggesting that these genes occurred in a common ancestor before *Chlamydomonas *[4,6]. Putative cytosolic OsCYP18-4, -19-2, and -19-3; secretional OsCYP19-4 and OsCYP21-1; mitochondria-targeting OsCYP20-1 and OsCYP22; and chloroplast-targeting OsCYP20-2 and OsCYP20-3 are closely related, with similar amino acid sequences, and mostly belong to 2 branches of clade I (Figure 6). Among them, OsCYP18-4, OsCYP19-2, OsCYP19-3, OsCYP19-4, OsCYP20-1, OsCYP21-1 and AtCYP22 share a 5-to-8 amino acid insertion located between the α1 helix and β3 sheet (Figure 4). However, all of these members are perfectly conserved in the 7 amino acids for CsA binding, except for Os-CYP19-4 in which 121-tryptophan is changed to arginine (Table 2), suggesting that these are catalytically active isoforms. Based on these characteristics, there is a relatively clear one-to-one orthology between rice CYPs and *Arabidopsis *CYPs, but no orthologs corresponding to AtCYP18-3 and AtCYP19-1 were found in rice. It has been suggested that two pairs of *Arabidopsis *CYPs, AtCYP18-3 and AtCYP19-1, and AtCYP18-4 and AtCYP19-2, arose by gene duplication, implying that the gene duplication occurred only in the *Arabidopsis *lineage because of divergence of the lineage between *O. sativa *and *Arabidopsis*, and these two pair of genes may entail redundancy [15,46]. Another cytosolic OsCYP19-3 that shows 79% identity with AtCYP19-3 also is very similar to OsCYP19-2 in amino acid sequence (Additional file 1), except for some divergence in the α3 domain (Figure 4). The OsCYP19-3 may have a distinct function from OsCYP19-2, considering that OsCYP19-3 has been survived a long period of evolution in most plants, including *Arabidopsis *and rice (data not shown). It is also widely accepted that gene duplication is a primary source of new functional genes [47]. Putative ER targeting SD OsCYPs, OsCYP19-4 and -21-1 show 70 and 69% similarity to the ER proteins AtCYP19-4 and AtCYP21-1, respectively. They also display 68 and 69% similarity with putative ER localized CYN20-1, respectively, probably indicating an ancestral gene (Additional file 1). The ER location of AtCYP19-4 (CYP5) has been confirmed by N-terminal green fluorescent protein fusion, and may involve the modulation of a guanine nucleotide exchange factor essential for vesicle trafficking [48,49]. Interestingly, OsCYP20-1 has the greatest similarity to putative ER located AtCYP20-1 and CYN20-1; however, it showed mitochondrial localization by both TagetP and PSORT analysis. Unlike AtCYP20-1, OsCYP20-1 retains additional 21-mer (maybe a signal for mitochondrial targeting) at the N-terminus of the protein (Figure 4), and this additional peptide proved to be common in several other plants (data not shown). Thus, the exact location of OsCYP20-1 needs to be determined by further experiments. In particular, AtCYP21-1, the ortholog of OsCYP21-1, has yet to have its location verified, being either cytosolic or ER [4,50]. In the meantime, rice lacks the ortholog for another ER CYP, AtCYP21-2, suggesting that AtCYP21-2 was duplicated from one of the other ER targeting isomeric genes: AtCYP19-4, -20-1, or -21-1, in the *Arabidopsis *lineage alone. Among OsCYPs that have highly conserved CsA binding amino acids, OsCYP20-2 (chloroplast thylakoid lumen) and OsCYP20-3 (chloroplast stroma) show ortholog relationships with AtCYP20-2 and AtCYP20-3 and CYN20-3, respectively. However, OsCYP22, the ortholog of AtCYP22 and CYN22 that are putatively cytosolic, scored high for targeting to chloroplast stroma based on both PSORT II and Target P analysis. AtCYP22 also contains an N-terminal extension, but it did not score highly for targeting to any subcellular compartment, whereas the signal peptide of AtCYP22 shows the changes in some amino acids containing the insertion of hydrophobic 5 amino acids, which may affect its signaling properties (data not shown).

**Table 2:** Conservation of key residues for Cyclosporin A binding/PPIase activity in *O. sativa *cyclophilins (OsCYPs) and comparison with those of *A. thaliana *cyclophilins.

OsCYPs/AtCYPs		Residues for Cyclosporin A binding/PPIase activity in human cyclophilinA (aa position)							Conserved^a^ (%)	Similarity^b^ (%)
				
		54	55	60	111	113	121	126		
hCYPA		H	R	F	Q	F	W	H		
OsCYP17		S	Q	I	"	"	"	Y	43/-	
OsCYP18-1	: AtCYP18-1	"	"	"	"	"	H	Y	71/71	100
OsCYP18-2	: AtCYP18-2	"	"	"	"	"	S	"	86/86	100
	: AtCYP18-3	"	"	"	"	"	"	"	-/100	
OsCYP18-4	: AtCYP18-4	"	"	"	"	"	"	"	-/100	
	: AtCYP19-1	"	"	"	"	"	"	"	-/100	
OsCYP19-2	: AtCYP19-2	"	"	"	"	"	"	"	100/100	100
OsCYP19-3	: AtCYP19-3	"	"	"	"	"	"	"	100/100	100
OsCYP19-4	: AtCYP19-4	"	"	"	"	"	R/"	"	88/100	88
OsCYP20-1	: AtCYP20-1	"	"	"	"	"	"	"	100/100	100
OsCYP20-2	: AtCYP20-2	"	"	"	"	"	"	"	100/100	100
OsCYP20-3	: AtCYP20-3	"	"	"	"	"	"	"	100/100	100
OsCYP21-1	: AtCYP21-1	"	"	"	"	"	"	"	100/100	100
	: AtCYP21-2	"	"	"	"	"	"	"	-/100	
	: AtCYP21-3	S	"	K	E	"	D	L	-/29	
OsCYP21-4	: AtCYP21-4	R/Q	H/"	F/Y	D/E	L	D	L	0/14	43
OsCYP22	: AtCYP22	"	"	"	"	"	"	"	100/100	100
OsCYP23	: AtCYP23	F	"	"	S	S	H	Y	29/29	100
	: AtCYP26-1	D	H	L	"	O	Q	"	-/29	
OsCYP26-2	: AtCYP26-2	V	K	Y	E	V	E	A/V	0/0	86
OsCYP28	: AtCYP28	"	K	Q	E	L	E/Q	N	14/0	86
OsCYP37	: AtCYP37	Y	T	L	"	"	F	F	29/29	100
OsCYP38	: AtCYP38	F	"	"	"	"	I	Y	57/57	100
OsCYP40a	: AtCYP40	"	"	I	"	"	H	"	71/71	100
OsCYP40b	: AtCYP40	"	"	I	"	"	H	"	71/71	100
OsCYP57	: AtCYP57	"	"	"	"	"	"	N/"	86/100	86
OsCYP59a	: AtCYP59	R/Q	K	Q	"	Y	Y	"	29/29	86
OsCYP59b	: AtCYP59	R/Q	K	Q	"	Y	Y	"	29/29	86
OsCYP63	: AtCYP63	"	F/"	M/"	"	"	H	"	57/86	71
OsCYP65	: AtCYP65	"	"	"	"	"	H	"	86/86	100
OsCYP71	: AtCYP71	"	"	"	"	"	"	"	100/100	100
OsCYP95	: AtCYP95	"	"	"	"/H	"/H	R/Q	S/N	57/43	43
Conserved^c ^(%)		71/77	79/79	72/69	82/79	71/71	38/52	62/69		

^a^% identity of rice/*Arabidopsis *CYP compared to human cyclophilin A. ^b^% similarity between rice and *Arabidopsis *CYP. ^C^% conservation of amino acid in rice/*Arabidopsis *CYP

Among SD CYPs showing significant phylogenic diversity in amino acids for CsA binding mitochondria-located, OsCYP21-4 is not conserved in 7 amino acids for PPIase and shows 54% identity in the amino acid sequence with AtCYP21-4. It also does not have an ortholog in *Chlamydomonas*, suggesting it appeared after *Chlamydomonas *(Additional file 1 and Table 2). Rice lacks the ortholog for another mitochondria CYP, AtCYP21-3, which shows very similar signal peptides for 47 amino acids and highly homologous C-terminal CYP domains with AtCYP21-4, suggesting that it originated by duplication of AtCYP21-4 only in the *Arabidopsis *lineage [4]. ER-located OsCYP23 is also significantly divergent in key residues for CsA binding, but it has orthologs in other plants (data not shown) containing both *Arabidopsis *and *Chlamydomonas*, which suggests that it has a distinct function with ER-located other CYPs in which PPIase activity is well conserved (Table 2). Chloroplast-located SD CYPs OsCYP26-2, -28, -37 and -38 show a very low conservation ratio and have significant modifications in key residues (Figure 4). They also belong to clade III, based on phylogenetic analysis (Figure 6), unlike AtCYP20-2, which is present in clade I and has highly conserved key residues for CsA binding [51-53]. Although they show high divergence in their amino acid sequences, including the CYP area, they have orthologs with significant homology in the CYP area, including key residues in both *Arabidopsis *and *Chlamydo-monas*. The diversity may be inherited from cyanobacteria, which contains two CYPs similar to CYN37 and -38 [6]. The putative leucine-zipper domain of AtCYP38 (TLP40) is not conserved in OsCYP38, CYN38 and orthologs of other plants (data not shown), and the role of the domain has not been clarified after several studies, indicating that this is not a conclusive point of evolution for the proper function of this protein [54-56]. All chloroplast lumen-located OsCYPs also contain double arginine residues, and follow the hydrophobic region needed for chloroplast lumen targeting (the signal sequences of OsCYP37 and OsCYP38 are not shown here for adequate CYP domain alignment) in the extension signal of the N-terminus (Figure 4).

Nine OsCYPs belong to MD members. Among them, two putative cytosolics, OsCYP40 (a/b), contain the C-terminal TPR domain and show 73 and 70% similarity to AtCYP40, respectively (Additional file 1). The existence of two isomeric genes has even been confirmed in some plants (*Poplus*, *Sorghum *and *Vitis*) through BlastP analysis (data not shown). Duplication probably occurred immediately after the rice and *Arabidopsis *lineages separated. The putative nuclear-located OsCYP59a and -59b, seemingly representing another duplicated gene among MD OsCYPs, are organized in tandem on the same chromosome (Additional file 1), and have RRM, zinc-finger and Arg/Lys-rich domains (Figure 5). They are orthologous to AtCYP59, which was identified as a dynamic interplayer between transcription and pre-mRNA processing through interaction with SR proteins and the C-terminal domain of RNA polymerase II [57]. Interestingly, only OsCYP59a includes the long insertion between the α1/β3 and β4/β5 domains, and they may have distinct functions. However, only one form of two isomeric genes was found in the other plants (data not shown), indicating that the duplication occurred most recently within the rice lineage. Nuclear-located OsCYP57 and OsCYP65 and cytosolic OsCYP71 belong to clade II (Figure 6) and have well conserved CYP domains (Table 2). These are orthology relationships with CYPs with the same suffix number for AtCYP and CYN. OsCYP65 contains the U-box and Arg/Lys-rich domain, and was classified as putatively nuclear targeting, unlike cytosolic AtCYP65, for which no Arg/Lys-rich domain was located (Figure 5). OsCYP71 contains the N-terminal WD40 repeats and shows high orthology (81% identity) with AtCYP71 (Additional file 1). Finally, putative nuclear-located MD OsCYP95 is annotated to 436 amino acids in the GRAMENE database and 670 amino acids in the NCBI database, respectively, although they are proteins from the same loci of chromosome II. From the analysis of other plant CYPs containing *O. sativa *ssp. Indica using the GRAMENE and NCBI database, we conclude that the 670 amino acid from the NCBI database is probably correct. The ortholog of OsCYP95 is absent in *Chlamydomonas*, and belongs to the same branch as OsCYP63 within clade II (Figure 6), indicating that OsCYP95 may be a duplicate of OsCYP63 from before the divergence of rice and *Arabidopsis*, but after *Chlamydomonas*.

### Expression Patterns of Immunophilins under Salt and Desiccation Stress

**Figure 7:**
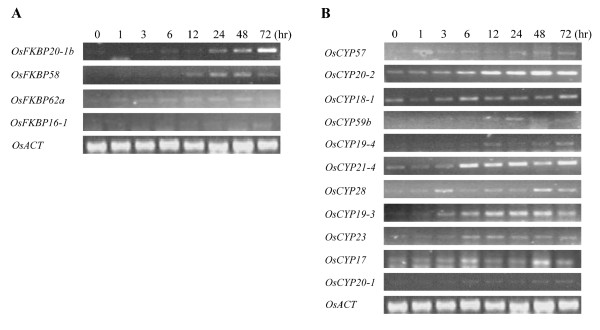
**Expression level of representative immunophilins under salt stress**. Semi-quantitative RT-PCR was carried out with gene specific primers using cDNAs from RNA samples. RNAs were isolated from two-week-old seedlings were exposed to 200 mM NaCl and tissues were harvested at the indicated time points. The rice actin level was used for control. A, OsFKBPs RT-PCR analysis; B, OsCYPs RT-PCR analysis.

The expression of most of rice's IMMs was identified by the presence of ESTs in the NCBI *O. sativa *EST database. OsFKBP44 and OsCYP17 did not show ESTs, but their expression was confirmed by RT-PCR analysis (Additional file 1). The expression level using the latter method corresponds roughly to the number of ESTs with all tissues of young plantlets (data not shown). To identify stress-induced Os IMMs under salt and desiccation conditions, RT-PCR was carried out at least 3 times on each IMM. Under the salt stress of 200 mM NaCl, OsFKBP20-1b, -58, -16-1, and -62a mRNA levels were increased. The level of gene expression began to increase 24 h after salt treatment, but in the case of OsFKBP62a it rose 1 h after salt treatment. The strongest salt induction pattern can be seen on OsFKBP20-1b, which is rarely expressed in the absence of stress (Figure 7A). In the OsCYPs, OsCYP19-3, -20-2, -21-4, -23, and -28, gene expression was strongly induced 3 h after salt treatment and continued until 72 h. OsCYP17 -19-4, -20-1, -57, and 59b expression was also induced under salt stress. In particular, OsCYP59b expression increased only 24 h after treatment (Figure 7B).

**Figure 8:**
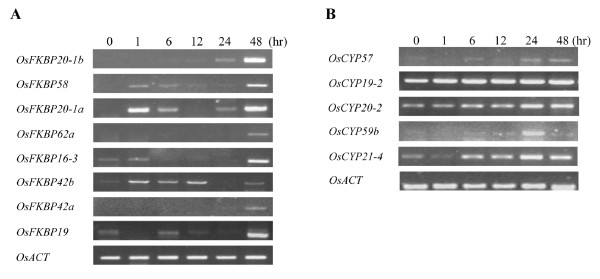
**Expression level of representative immunophilins under desiccation stress**. Semi-quantitative RT-PCR was performed with gene specific primers using cDNAs from RNA samples. RNAs were isolated from two-week-old seedlings were exposed to drought stress and tissues were harvested at the indicated time points. The rice actin level was used for control. A, OsFKBPs RT-PCR analysis; B, OsCYPs RT-PCR analysis.

Regarding desiccation stress, 8 OsFKBPs and 5 OsCYPs were upregulated in our water stress conditions (see the methods). Expression in OsFKBP19, -20-1a, and -20-1b was strongly induced by desiccation stress, with OsFKBP16-3, -42a, -42b, -58, and -62a also showing increased expression levels. Expression in OsFKBP20-1a, -58, and -42b was induced at 1 h after desiccation stress, but expression in OsFKBP16-3, -19, -20-1b, -42a, and OsFKBP62a was induced 24 h after desiccation (Figure 8A). In OsCYPs, the expression of the OsCYP21-4 and -59b genes were induced significantly under desiccation stress, whereas expression for OsCYP19-2, -20-2, and -57 was only slightly increased. Among the desiccation affected OsCYPs, OsCYP19-2, -20-2, -21-4, and -57 showed increased expression 6 h after stress, but only OsCYP59b showed a change in the induced expression at 24 h after desiccation, being notably similar to salt stress response (Figure 8B). As with OsCYPs induced under salt stress, desiccation inducing OsCYPs also are composed of CYPs targeted to the various cellular organs. These results suggest that various subcellular Os IMMs respond to abiotic water-stress (salt and desiccation) to defend or regulate cellular effects under changed circumstances. Some of the Os IMM genes were upregulated by both salt and desiccation stress, but others showed gene expression against only one stress. This suggests that although both salt and desiccation cause water stress to cells in common, some IMMs may be involved in distinct signal transduction or defense pathways against each individual stress before the cell activates a common stress response.

Three OsFKBPs, OsFKBP20-1b, -58 and 62a, were upregulated under both salt and desiccation stress. *Arabidopsis *ROF1 (AtFKBP62) expression was induced by heat stress and developmentally regulated. ROF1 binds heat-shock proteins HSP90.1 via the TPR domain and is located in the cytoplasm under normal conditions, but in the nucleus during heat stress [19]. The expression of rice IMM FKBP62a (rFKBP64) was also elevated by heat stress in various organs, indicating that OsFKBP62a plays specific physiological functions in other stress responses as well as in the heat stress response in plants [40]. As stated above, the putative nucleus OsFKBP58 (OsFKBP53a) lacks an ortholog in *Arabidopsis*, and only the OsFKBP58 gene responded to both salt and desiccation stress, suggesting that it plays a different role from OsFKBP53 and AtFKBP53.

Expression in OsFKBP16-3, 19, -20-1a, -42a, and -42b was upregulated under desiccation, but not salt, stress. OsFKBP16-3 contains two cysteine residues separated by 3 intervening non-cysteine residues at the N-terminus of the putative mature protein, predicting an intra-disulfide bond and suggesting a role for the redox response related to stress. Actually, the activity of a stromal AtCYP20-3 (ROC4) and the thylakoidal AtFKBP13, are regulated by redox relay of the thiol group [58,59]. Another thylakoid IMM, OsFKBP19, which has two cysteine residues in the loop regions between the β2 and β3 domains, is also upregulated by desiccation stress, whereas thylakoid lumen OsFKBP13 and OsFKBP16-2 that have two coupled cysteine residues forming a disulfide bond and upregulated by light irradiation, did not respond to salt and desiccation stress [4]. OsFKBP20-1a proved to be nuclear-targeting and heat-inducible; its over-expression in yeast indicates a capacity for high-temperature tolerance [60]. They also show that the OsFKBP20-1a and SUMO-conjugating enzyme interacted physically to mediate the stress response of rice plants. The paralog OsFKBP42a and 42b are ~90% identical, but they have slightly different EST numbers (Additional file 1) and distinct spatial expression profiles, with OsFKBP42a being expressed at the highest levels in crown tissue, whereas OsFKBP42b is expressed more highly in the roots [8]. Expression of both OsFKBP42a and -42b was upregulated by desiccation, but the timing of the increase for both genes was different. Taken together, the two paralog genes OsFKBP42a and -42b may retain the same function, but have different spatial/temporal regulation.

Among the 9 OsCYP genes upregulated under salt and desiccation stress, 4 genes (OsCYP20-2, -21-4, -57, and -59b) responded to both forms of stress, while others responded only to salt stress. AtCYP20-2, the ortholog of OsCYP20-2, the only CYP to show PPIase activity in the thylakoid lumen, was increased when plants were exposed to strong light or low temperature, and it has already been suggested that this PPIase plays a functional role in acclimation responses [52,61,62]. However, the phenotype and protein composition of the thylakoid lumen in *AtCYP20-2 *mutants seems indistinguishable from that of the wild type plants, and it is concluded therefore that AtCYP20-2 is unrelated to its PPIase capacity [52,53]. The OsCYP20-2 gene, the ortholog of AtCYP20-2, is also expressed at the highest level in the OsCYP family (Additional file 1), showing distinct upregulation under both salt and desiccation stress. From these results, we suggest that At/OsCYP20-2 maintains the homeostatic state of thylakoid lumen under stress, although it may be unrelated to its PPIase activity. Another two CYPs, the putative mitochondrial OsCYP21-4 and nuclear OsCYP57, were positively regulated in response to both salt and desiccation stress; none of the functions of the two proteins and their other plant orthologs are known. Nuclear-located AtCYP59b with the *Arabidopsis *ortholog OsCYP59b and the RNA-recognition motif (RRM), interacted with the C-terminal domain of the largest subunit of RNA polymerase II and SR proteins, and this may function in activities connecting transcription and pre-mRNA processing [57]. The OsCYP59b gene was transiently upregulated only 24 h after both stress treatments; however, OsCYP59a (maybe a duplicate of OsCYP59b) expression was unchanged by salt/desiccation stress.

The expression of 5 OsCYPs (OsCYP19-3, -19-4, 20-1, -23, and -28) was upregulated by salt, but not by desiccation, stress. Cytosolic AtCYP18-1, -18-2, -18-3, -18-4, -19-1, -19-2, and -19-3 are highly conserved and probably have similar functions [4]. Similarly, cytosolic OsCYP19-2, -19-3, and -19-4 are highly conserved; OsCYP19-2 expression was upregulated by desiccation stress, and OsCYP19-3 and OsCYP19-4 were upregulated by salt stress, suggesting that these genes also have a similar function. The ortholog of OsCYP19-4, AtCYP19-4 (CYP5), interacts with GNOM, which is involved in the coordination of cell polarity along the apical-basal embryo axis in *Arabidopsis *[48]. OsCYP19-4 was abundant only in pistils at anthesis and 1 h after anthesis and was undetectable in leaves, roots, flowers, and embryos. CYP5 may regulate the function of the GNOM protein during embryogenesis [63]. However, considering our results indicating that the OsCYP19-4 gene was expressed in young rice plants without pistils or in the embryogenesis stage under salt stress, the full role of this protein remains to be clarified. AtCYP20-1, the ortholog of OsCYP20-1, interacted with the regulatory subunit RCN1 of a Ser/Thr-specific protein phosphatase, which suggests that AtCYP20-1 and the RCN1 complex is required for the control of root cell proliferation [64]. AtCYP20-1 was identified as an unfolded protein response (UPR) gene following endoplasmic reticulum stress [65]. Unlike with AtCYP20-1, nothing about the function of the putative mitochondria locating OsCYP20-1 has been reported. Putative ER-locating OsCYP23 and thylakoid lumen-locating OsCYP28 are not conserved in amino acids for CsA binding (Table 2), and given their upregulated expressions only under salt stress, but not under desiccation stress, we must infer that they are associated with specific substrates for metabolic regulation related to sodium or chloride ions.

### Tissue Expression Profiles of Stress inducible Rice Immunophilins

**Figure 9:**
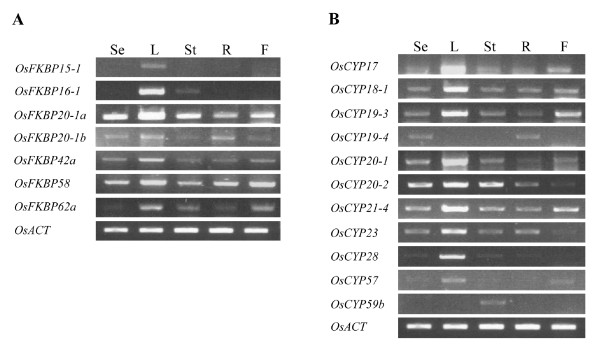
**Expression levels of some immunophilins (especially, showing changes in mRNA expression levels under water stress) in different tissues**. Semi-quantitative RT-PCR was performed with gene specific primers using cDNAs from RNA samples. RNAs were isolated from RNA samples isolated from different tissues. The tissues were used 6 days growing seedling (Se), leaves (L), stems (St), roots (R) and flowers (F). The rice actin level was used for control. A, OsFKBPs RT-PCR analysis; B, OsCYPs RT-PCR analysis.

The EST database and our reverse transcription PCR analysis indicate that most putative OsIMMs genes are expressed in plants. The transcript levels of some of the stress inducible OsIMMs have been investigated in different tissues (Figure 9). The analysis indicated many of the genes (OsFKBP 20-1a, -20-1b, -58, -42a, and OsCYP18-1, -21-4, -19-3, -17 and -20-1) are expressed in all tissues, including 6-day seedlings, leaves, stems, roots and flowers, in agreement with previous *Arabidopsis *orthologs results [4]. But some of the stress-inducible OsIMMs (OsFKBP15-1, -16-1, -62a, and OsCYP19-4, -28, -57, -59b) are not consistent with the *Arabidopsis *orthologs results: OsFKBP15-1 is expressed only weakly in leaf, compared to the AtFKBP15-1 which is expressed strongly in all tissues. OsFKBP16-1 is expressed only in leaves and stems, but the AtFKBP16-1, a chloroplast lumen protein, is well expressed in most of tissues except roots. OsFKBP62a is expressed in leaves, stems and flowers, but not in seedlings and is extremely low in roots, whereas AtFKBP62 is expressed in all tissues. OsCYP19-4 is expressed at very low levels only in seedling and roots, whereas AtCYP19-4 was expressed in all tissues. OsCYP28 is only expressed in leaves, whereas AtCYP28, a thylakoid lumen protein, is well expressed in all tissues except roots [4]. OsCYP57 is only expressed in leaves and flowers at low levels, compared to the high expression of AtCYP57 in all tissues. Finally, OsCYP59b is a rare case of expression pattern in which transcripts can be detected only in the stem at very low levels, but AtCYP59 is at high levels in all tissues. These results suggest that even if they are orthologs, each orthologous IMM in other organisms are under a different spatial/temporal-regulation as a result of environmental or physiological adaptation.

### Subcellular Localization of Os Immunophilins in Plant Cell

**Figure 10:**
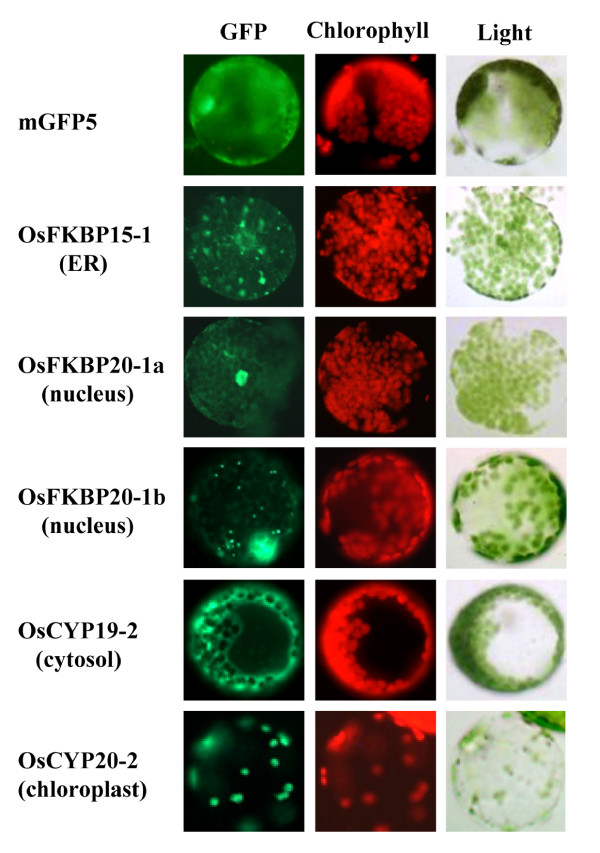
**Subcellular localization of *O. sativa *immunophilins using GFP fusion proteins**. The full length ORF of Os immunophilins FKBP15-2, FKBP20-1a, FKBP20-1b, CYP19-2 and CYP20-2 were fused to the N-terminal mGFP5 in pCAMBIA1302 and expressed in *N. benthamiana *leaf cell transformed using *Agrobacterium*. Localization of fluorescent signals was examined at 48 h after *Agrobacterium *infiltration under fluorescence microscopy. The images of GFP, chlorophyll auto-fluorescence and bright-field images are shown.

According to the prediction database, OsFKBP15-1 is localized to ER; OsFKBP20-1a and -b are localized to nucleus (Figure 2);OsCYP19-2 is cytosolic; and OsCYP20-2 is located in the chloroplast lumen (Figure 5). To access the validity of subcellular localization for predicted Os IMM proteins, OsFKBP15-1, -20-1a, -20-1b, OsCYP19-2 and -20-2 were fused with N-terminal GFP protein and then expressed in *N. benthamiana*, using *Agrobacterium *transiently (Figure 10). The control of mGFP5 protein fluorescence was accumulated in both cytosol and nucleus in tobacco cells. The GFP fusion proteins of OsFKBP20-1a and -b, predicted to be nuclear due to the C-terminal NLS sequences, were distinctly localized within the nucleus. However, OsFKBP20-1a GFP protein showed a typical nuclear targeting pattern, but the OsFKBP20-1b GFP proteins were strongly accumulated in a specific region within the nucleus and also in cytosol foci. The co-localization of OsFKBP20-1a and -b GFP proteins was confirmed by DAPI staining of the nucleus (data not shown). As the OsCYP19-2 sequence has not any a signal peptide for subcellular localization (according to the database), the GFP fusion protein was also located in the cytosol. OsCYP20-2 GFP protein, which contains double arginine residues and hydrophobic regions as a putative chloroplast lumenal protein, was clearly localized in chloroplasts. As a putative ER-targeting protein, OsFKBP15-1-GFP protein seemed to be localized in the ER, but we were unable to use a specific marker protein in this study. From these results, we suggest that although not all the putative localization of Os IMMs could be checked, *in silico *prediction programs for subcellular localization may useful for functional study of Os IMMs.

## Conclusions

Like the two green photosynthetic organisms, *Arabidopsis *and *Chlamydomonas*, in which putative IMM genes have been identified within the genome, rice has the largest number of putative IMM genes (29 FKBPs and 27 CYPs) among organisms that have been studied to date, showing that the diversity of IMM in green photosynthetic organisms was already present in the ancient green lineage, *Chlamydomonas*, and that this diversity is maintained in most land plants. In addition, the large number of IMM genes in the green lineage can be classified according to an ortholog relation. Specifically, most of the genes survived without degeneration or great modification during evolution, which suggests that most orthologous IMM genes may have a conserved function in cell metabolism. However, some rice IMM genes are clearly different from those of *Arabidopsis *and *Chlamydomonas*, and they may therefore have evolved and developed a distinct role along a different evolutionary lineage. In particular, 11 OsFKBPs and 7 OsCYPs of rice IMMs are putatively targeted to chloroplasts, as with *Arabidopsis*, in which 11 AtFKBPs and 5 AtCYPs are targeted to chloroplasts. Furthermore, most of the chloroplast IMMs in both plants has a putative ortholog relation, which indicates that they may have a conserved function in relation to photosynthesis. Also by investigating the expression of all OsFKBPs and OsCYPs under salt and desiccation stress conditions, we found that a significant number of genes were up-regulated in response to water stress. Furthermore, the tissue specific expression of water stress responding Os IMMs and the visual localization of some of these IMMs were examined. Ultimately, the classification of putative FKBPs and CYPs in rice provides information about the evolutionary/functional significance of each FKBP and CYP based on a comparison with the relatively well studied *Arabidopsis *and *Chlamydomonas *IMMs. The knowledge that the large numbers of IMM genes were upregulated by water stress offers the possibility of manipulating genes related to stress-related in rice research.

## Methods

### Sequence Analysis

Putative IMMs of *Oryza sativa *cv. Japonica were identified via BLAST searching of the Rice Genome Annotation Project database http://rice.plantbiology.msu.edu using the amino acid sequence of AtFKBP12 for FKBPs and AtCYP18-1 for CYPs as the query. The expressed sequence tags (ESTs) for individual rice IMM were counted by tBLASTn searches. The alignments of amino acid sequence were performed using ClustalW and displayed with Genedoc v.2.6.002 [66,67]. The phylogenetic tree was constructed by ClustalW using the above aligned amino acid sequence. To search for functional domains, SMART http://smart.embl-heidelberg.de, PROSITE http://us.expasy.org/tools/scanprosite, and a CaM database search http://calcium.uhnres.utoronto.ca were used. The protein targeting signal and subcellular location of the IMMs were predicted using several programs, such as PSORT http://psort.ims.u-tokyo.ac.jp, TargetP http://www.cbs.dtu.dk/services/TargetP/, SignalP http://www.cbs.dtu.dk/services/SignalP/, ChloroP http://www.cbs.dtu.dk/services/ChloroP/, and PredictNLS http://www.predictprotein.org/ for cross-confirmation. The identification of orthologous *Arabidopsis thaliana *and *Chlamydomonas reinhardtii *PPIase for individual rice IMMs were performed using BLASTP http://www.ncbi.nlm.nih.gov/BLAST/.

### Plant Growth and Treatment

Rice (*Oryza sativa L*. japonica genotype Dongjin) seeds were germinated and grown in Yoshida nutrient solution, which consists of NH_4_NO_3 _(1.43 mM), NaH_2_PO_4_.2H_2_O (0.37 mM), K_2_SO_4 _(0.5 mM), CaCl_2 _(1.0 mM) and MgSO_4_.7H_2_O (1.6 mM) [68]. Plants were grown in a controlled chamber with a photoperiod of 12 h light at 28°C and 12 h dark at 22°C. For the salt stress treatment, two-week-old rice seedlings were subjected to 200 mM NaCl and harvested at time courses of 0, 1, 3, 6, 12, 24, 48 and 72 hours after treatment. For the desiccation stress treatment, two-week-old rice seedlings were transferred to dry pots and harvested at 0, 1, 6, 12, 24 and 48 hours after the treatment time points. Different tissues of the rice (6-day-old seedlings, leaves, stems, roots and flowers) were collected and frozen in liquid nitrogen for RNA extraction.

### Total RNA Isolation and cDNA Synthesis

Total RNA was prepared from the two-week-old rice seedlings treated with salt or drought stresses using Trizol reagent (Invitrogen Life Technologies) according to the manufacturer's instructions. RNA was purified further using Qiagen RNeasy columns (Qiagen Inc., CA, USA) and on-column DNase treatment. Two μg of RNA were transcribed reversely in a 20 μl reaction volume using oligo dT 14 to 18 primer (Invitrogen Life Technologies) and reverse transcriptase (SuperScript III, Invitrogen Life Technologies). Reactions were incubated at 65°C for 5 min, 50°C for 50 min, and then 85°C for 5 min.

### RT-PCR Analysis

The resulting cDNA was used as templates in 20 μL of PCR reaction using specific primers. The gene-specific primers used for OsFKBPs and OsCYPs are described in Additional file 2 and 3: Supplemental Table S2 and S3. A total of 10 μL of PCR samples was separated by agarose gel electrophoresis and visualized with ethidium bromide staining. The expression of an actin gene was used as an internal control for determining the RT-PCR amplification efficiency among different reactions. The RT-PCR reactions were repeated three times and representative results from one experiment were shown.

### Subcellular localization

The OsIMMs FKBP15-1, FKBP20-1a and FKBP20-1b, CYP19-2, and CYP20-2 cDNA fragments corresponding to the 154, 186, 185, 171, 247, and amino acids were cloned into the pCAMBIA1302 plasmids using *Bgl*II and *Spe*I sites to generate the OsIMMs-GFP fusion proteins respectively. The various GFP constructs were introduced into *N. benthamiana *epidermal leaves by *Agrobacterium *infiltration [69]. Expression of the fusion proteins were monitored at 48 h after *Agrobacterium *injection by an Axiophot fluorescence microscope (Zeiss, Germany). The filter set were used (excitation 450-490 nm, emission 500-550 nm) and Texas Red (excitation 530-585 nm, emission 650 nm longpass) for green fluorescent proteins and autofluorescence of chlorophyll, respectively.

## Authors' contributions

HC and HP conceived the intellectual design of the project and wrote the manuscript. JA carried out most of sequence analysis to classify rice IMM and build up the manuscript. DK performed rice genome database search and sorting the IMM gene family. YY and MS analyzed semi-quantitative RT-PCR for water stress gene expression. BK and HH prepared salt and drought stress rice sample for RT-PCR analysis and also participated in part of the manuscript writing, the method section. JP gave suggestions for the manuscript writing. SL helped to coordination and completion the manuscript. All authors read and approved the final manuscript.

## Supplementary Material

Additional file 1 **Immunophilins in *O. sativa***. ^a^Names used in other literature: 1, Gollan and Bhave, 2009; Magiri et al., 2006. ^b^Rice genome initiative nomenclature was pro-vided by Gramene database. ^c^Predicted localization and prediction obtained using TargetP http://www.cbs.dtu.dk/services/TargetP/, ChloroP http://www.cbs.dtu.dk/services/ChloroP/ and PredictNLS http://www.predictprotein.org: TL, thylakoid lumen; S, stroma; ER, endoplasmic reticulum; Vac, vacuole. ^d, e^Isoform number and amino acid were predicted by the Gramene database http://www.gramene.org/. ^f^EST number was estimated by the tblastN http://www.ncbi.nlm.nih.gov/. ^g, h^The Mr and isoelectric point of the full-length/mature protein were predicted by Compute pI/Mw tool http://www.expasy.ch/tools/pi_tool.html. ^i^The confirmation of expression and expression intensity by RT-PCR analysis: Y, yes; N, no; (s), strong; (i), intermediate; (w), weak. ^j, k^Orthologous immunophilin and % identity (ID) were analysed by blastp http://www.ncbi.nlm.nih.gov/ of the NCBI database of the complete proteome of *A. thaliana *and *C. reinhardtii*: IP, immunophilin. Click here for file

Additional file 2 **Primer sequences of OsFKBP genes for quantitative RT-PCR**. Click here for file

Additional file 3 **Primer sequences of OsCYP genes for quantitative RT-PCR**. Click here for file
