# Supplementary material for: Classification of rice (oryza sativa l. japonica nipponbare) immunophilins (fkbps, cyps) and expression patterns under water stress
Source: BMC Plant Biol. 2010 Nov 18;10:253. doi: 10.1186/1471-2229-10-253 (PMC3012604; doi:10.1186/1471-2229-10-253)
Supplement: Additional file 1 — Immunophilins in O. sativa. aNames used in other literature: 1, Gollan and Bhave, 2009; Magiri et al., 2006. bRice genome initiative nomenclature was pro-vided by Gramene database. cPredicted localization and prediction obtained using TargetP http://www.cbs.dtu.dk/services/TargetP/, ChloroP http://www.cbs.dtu.dk/services/ChloroP/ and PredictNLS http://www.predictprotein.org: TL, thylakoid lumen; S, stroma; ER, endoplasmic reticulum; Vac, vacuole. d, eIsoform number and amino acid were predicted by the Gramene database http://www.gramene.org/. fEST number was estimated by the tblastN http://www.ncbi.nlm.nih.gov/. g, hThe Mr and isoelectric point of the full-length/mature protein were predicted by Compute pI/Mw tool http://www.expasy.ch/tools/pi_tool.html. iThe confirmation of expression and expression intensity by RT-PCR analysis: Y, yes; N, no; (s), strong; (i), intermediate; (w), weak. j, kOrthologous immunophilin and % identity (ID) were analysed by blastp http://www.ncbi.nlm.nih.gov/ of the NCBI database of the complete proteome of A. thaliana and C. reinhardtii: IP, immunophilin. [file 1471-2229-10-253-S1.PDF]

Table S1. Immunophilins in *O. sativa*

Gene name (Other name [a])	RGI ID[b]	Predicted Localization[c]	Isoforms No.[d]	Amino Acid No.[e]	ESTs[f]	Mr (kD)[g]	pI[h]	Exprerssion (Level [i])	Arabidopsis IP (%ID[j])	Chlamydomonas IP (%ID[k])
FKBPs										
OsFKBP12	LOC_Os02g52290	Cytosol	1	112	102	12.1	8.21	Y (s)	FKBP12 (74%)	FKB12 (46%)
OsFKBP13	LOC_Os06g45340	Chloroplast (TL)	2	218/176	4	22/13.8	9.6/6.78	Y (w)	FKBP13 (65%)	FKB16-2 (50%)
OsFKBP15-1	LOC_Os09g32526	Secretion (ER)	2	154/110	28	16.3/14.1	5/5.04	Y (s)	FKBP15-1(81%)	FKBP15-1 (69%)
OsFKBP15-2	LOC_Os01g68710	Secretion (ER)	3	158/158/137	4	16.8/14.3	10.2/8.86	Y (s)	FKBP15-2 (76%)	FKBP15-1 (73%)
OsFKBP16-1	LOC_Os02g10590	Chloroplast (TL)	2	211/150	2	22.8/14.8	9.34/5.75	Y (w)	FKBP16-1 (74%)	FKB16-1 (43%)
OsFKBP16-2	LOC_Os02g51570	Chloroplast (TL)	1	230	22	23.5/14.8	9.05/8.93	Y (s)	FKBP16-2 (67%)	FKB16-2 (46%)
OsFKBP16-3	LOC_Os08g42850	Chloroplast (TL)	2	261/225/169	19	27.1/15.9	8.3/4.8	Y (i)	FKBP16-3 (76%)	FKB16-3 (58%)
OsFKBP16-4	LOC_Os07g09040	Chloroplast (TL)	1	243	11	25.7/18.2	9.63/9.32	Y (w)	FKBP16-4 (68%)	FKB16-4 (64%)
OsFKBP17-1	LOC_Os02g07220	Chloroplast (TL)	4	213	6	22.2	7.09	Y (i)	FKBP17-1 (71%)	FKB16-4 (33%)
OsFKBP17-2	LOC_Os03g50080	Chloroplast (TL)	1	252	10	26.2/15.5	8.2/4.9	Y (i)	FKBP17-2 (70%)	FKB17-2 (45%)
OsFKBP18	LOC_Os02g02550	Chloroplast (TL)	1	242	8	25.8/17.2	9.68/7.97	Y (w)	FKBP18 (68%)	FKB18 (48%)
OsFKBP19	LOC_Os07g04160	Chloroplast (TL)	2	258/237	11	28.3/21.1	9.2/5.29	Y (i)	FKBP19 (68%)	FKB19 (58%)
OsFKBP20-1a	LOC_Os05g38370	Nucleus	3	186/139/138	19	19.9	7.42	Y (s)	FKBP20-1 (77%)	FKBP15-1 (48%)
OsFKBP20-1b	LOC_Os01g62610	Nucleus	1	185	2	20.1	5.94	Y (i)	FKBP20-1 (76%)	FKBP15-1 (47%)
OsFKBP20-2	LOC_Os07g30800	Chloroplast (TL)	1	258	11	21.1/16.5	10.4/9.6	Y (w)	FKBP20-2 (72%)	FKB20-2 (60%)
OsFKBP42a	LOC_Os12g05090	Cytosol	1	370	15	42.2	5.58	Y (i)	FKBP42 (70%)	FKB42 (43%)
OsFKBP42b	LOC_Os11g05090	Cytosol	1	375	42	42.4	6.27	Y (w)	FKBP42 (70%)	FKB42 (42%)
OsFKBP44	LOC_Os09g01670	Cytosol	1	416	0	44.3	5.03	Y (w)	FKBP53 (43%)	FKB53 (57%)
OsFKBP46	LOC_Os01g38359	Cytosol	2	422/417	17	45.6	4.99	N	FKBP65 (44%)	FKB53 (33%)
OsFKBP53 (53b) [1]	LOC_Os09g12270	Nucleus	1	401	4	44.2	5.89	Y (w)	FKBP53 (53%)	FKB53 (44%)

Continued on following page

Table S1. Continued

**Gene name (Other name [a])**	**RGI ID[b]**	**Predicted Localization [c]**	**Isoforms No. [d]**	**Amino Acid No. [e]**	**ESTs [f]**	***Mr* (kD) [g]**	**pI [h]**	**Exprerssion (Level[i])**	***Arabidopsis* IP (% ID[j])**	***Chlamydomonas* IP (% ID[k])**
OsFKBP57	LOC_Os01g38180	Cytosol	1	517	4	57.4/55.2	5.25/5.36	N	FKBP65 (44%)	FKB62 (32%)
OsFKBP58 (53a[1])	LOC_Os04g36890	Nucleus	1	525	37	57.2	5.93	Y (i)	FKBP53 (36%)	FKB53 (60%)
OsFKBP59	LOC_Os09g01650	Nucleus	1	545	3	60.2	4.79	Y (w)	FKBP53 (43%)	FKB53 (48%)
OsFKBP62a (64[1, 2])	LOC_Os08g41390	Cytosol/nucleus	2	591/580	79	65.7	4.95	Y (i)	FKBP62 (77%)	FKB62 (41%)
OsFKBP62b (65[1, 2])	LOC_Os04g28420	Cytosol/nucleus	2	585/584	29	65.6	5.13	N	FKBP62 (75%)	FKB62 (41%)
OsFKBP62c (75[1, 2])	LOC_Os02g28980	Cytosol/ER	1	682	11	74.8	4.92	Y (i)	FKBP62 (73%)	FKB62 (42%)
OsFKBP72	LOC_Os03g25140	Cytosol/nucleus	1	632	35	70.4	5.21	Y (i)	FKBP72 (67%)	FKB99 (27%)
OsFKBP73	LOC_Os01g38229	Nucleus	4	653/652/422/422	45	73.4	5.08	Y (i)	FKBP62 (44%)	FKB62 (28%)
OsTIG	LOC_Os06g20320	Chloroplast (S)	1	537	17	59.9/51.4	4.94/4.85	Y (i)	TIG (58%)	TIG1 (25%)
Cyclophilins (CYPs)										
OsCYP17	LOC_Os02g02090	Cytosol	1	139	0	16.2	10.04	Y (i)	CYP19-1 (37%)	CYN19-2 (33%)
OsCYP18-1	LOC_Os06g04000	Cytosol	1	160	29	17.52	8.5	Y (i)	CYP18-1 (89%)	CYN18-1 (70%)
OsCYP18-2	LOC_Os08g44520	Cytosol	3	178/176/164	69	19.8	9.85	Y (w)	CYP18-2 (82%)	CYN18-2 (75%)
OsCYP18-4	LOC_Os10g06630	Cytosol	1	181	9	18.9	9.4	Y (w)	CYP18-4 (63%)	CYN19-2 (75%)
OsCYP19-2	LOC_Os02g02890	Cytosol	1	172	98	18.4	8.49	Y (s)	CYP19-2 (78%)	CYN19-2 (76%)
OsCYP19-3	LOC_Os09g39780	Cytosol	2	179/179	29	19.2	7.9	Y (i)	CYP19-3 (79%)	CYN19-2 (75%)
OsCYP19-4	LOC_Os06g49470	Secretion (Vac)	1	208	1	22.1/19.2	9.45/9.04	Y (w)	CYP19-4 (70%)	CYN20-1 (68%)
OsCYP20-1	LOC_Os06g49480	Mitochondria	3	225/220/190	56	24.1/20.1	10.4/9.26	Y (i)	CYP20-1 (85%)	CYN20-1 (78%)
OsCYP20-2	LOC_Os05g01270	Chloroplast (TL)	1	250	90	26.6/19.5	9.85/8.53	Y (s)	CYP20-2 (86%)	CYN20-3 (72%)
OsCYP20-3	LOC_Os01g18210	Chloroplast (S)	1	238	3	25.4/21.9	7.90/6.24	Y (w)	CYP20-3 (66%)	CYN20-3 (63%)
OsCYP21-1	LOC_Os09g36670	Secretion (ER)	1	215	1	23.4/21.3	8.62/7.26	Y (w)	CYP21-1 (69%)	CYN20-1 (69%)

Continued on following page

Table S1. Continued

Gene name (Other name [a])	RGI ID[b]	Predicted Localization[c]	Isoforms No.[d]	Amino Acid No.[e]	ESTs[f]	M_r (kD)[g]	pI[h]	Exprerssion (Level [i])	*Arabidopsis* IP/ (% ID[j])	*Chlamydomonas* IP (% ID[k])
OsCYP21-4	LOC_Os07g29390	Mitochondria	2	235	31	26.4/21.6	8.9/5.88	Y (s)	CYP21-4 (54%)	CYN20-3 (37%)
OsCYP22	LOC_Os03g59700	Chloroplast (S)	1	204	4	21.8/20.1	8.29/8.47	Y (w)	CYP22 (76%)	CYN22 (74%)
OsCYP23	LOC_Os11g38990	Secretion (ER)	3	235/235/235	33	25.8/23.3	7.25/6.74	Y (i)	CYP23 (76%)	CYN23 (57%)
OsCYP26-2	LOC_Os01g02080	Chloroplast (TL)	1	304	7	31.4//25.3	9.45/8.42	Y (w)	CYP26-2 (49%)	CYN26 (44%)
OsCYP28	LOC_Os08g19610	Chloroplast (TL)	4	327/327/327/253	9	35.9/28.1	7.58/5.45	Y (w)	CYP28 (56%)	CYN28 (43%)
OsCYP37	LOC_Os07g37830	Chloroplast (TL)	2	465/432	7	49.8/47.9	5.56/5.51	Y (w)	CYP37 (74%)	CYN37 (47%)
OsCYP38	LOC_Os08g29370	Chloroplast (TL)	2	426/427	21	46.6/39.7	4.6/4.54	Y (i)	CYP38 (72%)	CYN38 (54%)
OsCYP40a	LOC_Os06g11320	Cytosol	2	396/396	6	43.25	5.7	Y (i)	CYP40 (73%)	CYN40 (51%)
OsCYP40b	LOC_Os02g52360	Cytosol	2	403/403	3	44	5.28	Y (w)	CYP40 (70%)	CYN40 (48%)
OsCYP57	LOC_Os01g40050	Nucleus	2	499/499	19	56.9	6.19	Y (i)	CYP57 (58%)	CYN57 (55%)
OsCYP59a	LOC_Os06g45900	Nucleus	1	548	3	63.2	6.32	Y (w)	CYP59 (56%)	CYN59 (49%)
OsCYP59b	LOC_Os06g45910	Nucleus	1	564	31	65.05	5.77	Y (i)	CYP59 (58%)	CYN66 (61%)
OsCYP63	LOC_Os07g08190	Nucleus	1	632	3	70	11.21	Y (w)	CYP63 (67%)	CYN40 (59%)
OsCYP65	LOC_Os03g10400	Nucleus	3	586/462/462	9	64.7	8.36	Y (w)	CYP65 (72%)	CYN65 (51%)
OsCYP71	LOC_Os08g44330	Cytosol	2	651/513	6	72.9	6.94	Y (w)	CYP71 (81%)	CYN71 (52%)
OsCYP95	LOC_Os02g10970	Nucleus	3	436/436/436	2	49	10.38	Y (w)	CYP95 (61%)	CYN40 (61%)
