# Supplementary material for: Classification of rice (oryza sativa l. japonica nipponbare) immunophilins (fkbps, cyps) and expression patterns under water stress
Source: BMC Plant Biol. 2010 Nov 18;10:253. doi: 10.1186/1471-2229-10-253 (PMC3012604; doi:10.1186/1471-2229-10-253)
Supplement: Additional file 2 — Primer sequences of OsFKBP genes for quantitative RT-PCR. [file 1471-2229-10-253-S2.PDF]

Table S2 - Primer pairs used for RT-PCR analysis of OsFKBPs expression

Gene Name	Primer Sequences (F, forward; R, reverse[5’- 3’])	
OsFKBP12	F: atgggcttcgagaagacgatcctgaaag	R: ttactgggcgctaagaacctcaatctcg
OsFKBP13	F: atggctccctccgcccgccgcc	R: tcatccgactgctctgccgacgtactc
OsFKBP15-1	F: atggcgaagccgcagcttcttctgg	R: ctaaagctcgctgtcatcatcgtcc
OsFKBP15-2	F: atggggaagcgtcagcagcagcggcgg	R: ttagagctcactattgctagcaccacc
OsFKBP16-1	F: atggcgcccgcgtcgctgacgag	R: tcagaggaccttgagcagctgaac
OsFKBP16-2	F: atggctgcaaccgcttcgtcctcctcc	R: tcacttgtaaaatccaaccaggaagatg
OsFKBP16-3	F: atggctgcaaccgcttcgtcctcctcc	R: tcactcatcatcaagacctggtatgtac
OsFKBP17-1	F: atggtcagtctgtccgccgccgtcgctc	R: ttatgaatgctgccttatgctgattagc
OsFKBP17-2	F: atggccaccttccttgggagctcgccgg	R: tcacgccggcgcgatggacaccttgtc
OsFKBP18	F: atgcgcgccatttcggctcttctcc	R: tcatttctctgcaggagggacaac
OsFKBP19	F: atggacttggtgcgcgcgctcgccgcc	R: ctattgacttggtataatcttcaaaagctc
OsFKBP20-1a	F: atggcagaggttgcagatttaactggg	R: ctattttccctttccttttcccttcc
OsFKBP20-1b	F: atgtctgagaccatagatttaaccggag	R: ctatttggccttctttcccttgccctt
OsCYP20-2	F: atggagctggtctcctcgtcgcgctcc	R: tcagctgcacactacatcggagtaaaagg
OsFKBP42a	F: atggccgtgctggaagaagatccggcc	R: ctactcatccttgcgtttgaacagtttaac
OsFKBP42b	F: atggctgtggtggaggaagaagagggc	R: ctaggttggcctattgctggcacgctg
OsFKBP45	F: atgggcgggggcggagaaggcagc	R: tcatagctgaatgtaggctcatcc
OsFKBP46	F: atgccgatcccggttgatctgggc	R: tcagtttgcaggccatggcctg
OsFKBP53	F: atggcgttctggggtgtggaggtg	R: tcatgatgtgcttttggatctgacc
OsFKBP54	F: gaggagatgaagcagctcactttg	R: ataagccagctcaggaggaactatg
OsFKBP56	F: aggagatattacaaggccaggcag	R: tcagttacaatctgaagagatgcatc
OsFKBP58	F: catatccacactgatgctgaggaag	R: tcactttacattcacgagttccacatc
OsFKBP61	F: atggcgaccttctggggattggag	R: tcaacgtgcccttttggctcgcttc
OsFKBP62a	F: ctgcaacttgaacaatgctgcctgc	R: gagttaggcagcagtaacaggttccgatc
OsFKBP62b	F: tgcaagctcaacaatgctgcttgc	R: tcaagcggcactgtcaatggccatagg
OsFKBP62c	F: ggagcttgacagcatgaatgtgaag	R: gagttaacagttgatgcagtctacttcag
OsFKBP72	F: gagaagatgatagctgtcgacaag	R: tcagaccattgtgcacctgttgagccc
OsFKBP74	F: atgccgatctcgcatgagctgggcgag	R: tcacggctcggaatgtacaacggatg
OsTIG	F: atggagctcgccaccgccaccgcc	R: tcatggtctaatgtactcaataatg
