# Supplementary material for: Classification of rice (oryza sativa l. japonica nipponbare) immunophilins (fkbps, cyps) and expression patterns under water stress
Source: BMC Plant Biol. 2010 Nov 18;10:253. doi: 10.1186/1471-2229-10-253 (PMC3012604; doi:10.1186/1471-2229-10-253)
Supplement: Additional file 3 — Primer sequences of OsCYP genes for quantitative RT-PCR. [file 1471-2229-10-253-S3.PDF]

Table S3 - Primer pairs used for RT-PCR analysis of OsCYPs expression

Gene Name	Primer Sequences (F, forward; R, reverse[5’- 3’])	
OsCYP17	F: atggagctgtacgcggacctggtc	R: aatattagtagttaacgacggcggc
OsCYP18-1	F: atgtcggtgacgctgcacacgaatctc	R: ttagttggcgagagggttggcgtggatg
OsCYP18-2	F: atggatccaaggtcaaaagccgac	R: tcaatctttgaccacagtccgcagg
OsCYP18-4	F: atggcgccggcggcgtcctccaag	R: ctagagcacgccgcagtcggtg
OsCYP19-2	F: atgtcgaacacgagggtgttcttcga	R: ctaggagagctggccgcagtcggc
OsCYP19-3	F: atggcgagcaagaaccccaaggtgttc	R: tcagttggcgtggtcgtcggcgagctg
OsCYP19-4	F: atggcggcgagggagacgtcgcgccac	R: tcacttcagttcgccgctgtctgatatg
OsCYP20-1	F: atggcggggagcgggtggagg agg	R: ttacatcggcagttcgccgctgtcc
OsCYP20-2	F: atggcggtcgcaacctccttcgccacac	R: ttagaccactggaagctccccacactc
OsCYP20-3	F: atggcttgcaggccggccgtgtgc	R: tcaaccgtccacgggcagctcgcc
OsCYP21-1	F: atgctgcggaaggtggccgtcgccttc	R: ctacacttcttcagcccatttctccttcg
OsCYP21-4	F: atggcgaggataaagccgaagcaattg	R: tcagctcaaagcttgctgttttagcgtgatg
OsCYP22	F: atggcgtcctcggggggagcggcgatc	R: ctacatctcaccacactcgcttatgac
OsCYP23	F: atggcggctttgctccgccacgccgcg	R: tcatgcaaagcactttcttctccatctc
OsCYP26-2	F: accgagttcgtcatcaccaccggcgac	R: ctactgctcctgctgctgttcaatgac
OsCYP28	F: atggtgttgccttcatcaaataccagg	R: ttatggcagacttggaggggaaagtgatg
OsCYP37	F: atggcgtcgcgagcggcggccgcg	R: tcagctctctgccggcgcttctgg
OsCYP38	F: atggcggcggcgctcgcgttccccacctgc	R: ttatcctacaatcttatagcttgggttgg
OsCYP40a	F: gaagggtgctctgttggatgctg	R: tcacttgctctcttcattgtctttgtc
OsCYP40b	F: atggagggcggtggggagggcac	R: tcagttattctcttcatcgatcttctc
OsCYP59a	F: atgtcggttctcatagtgaccagc	R: tcatctcgtgtggcggttgctcct
OsCYP59b	F: atgtcggtcctcatcgtgaccagcgtg	R: ctatctatcatctctctgcctcctttcc
OsCYP63	F: gttcggtctcgctacaatgatggaag	R: tcagtatgaaaccaagcctttacttc
OsCYP65	F: cttatacattcaggaagaggtgttgtc	R: ctaccacccagagaaatccttaaat
OsCYP71	F: cccattgccggaatggttattatg	R: ttatgtctttggaactgtaacgttc
OsCYP95	F: atggcaaagaagaagaacccaattg	R: tcagatgaaacactgctcgtggaca
